# CVD-Engineered Nano Carbon Architectures: Mechanisms, Challenges, and Outlook

**DOI:** 10.3390/nano15231834

**Published:** 2025-12-04

**Authors:** Maria Hasan, Szymon Abrahamczyk, Muhammad Aashir Awan, Ondřej Sakreida, Alicja Bachmatiuk, Grazyna Simha Martynková, Karla Čech Barabaszová, Mark Hermann Rümmeli

**Affiliations:** 1 Electron Beam Emergent Additive Manufacturing (EBEAM) Centre, Centre for Nanotechnlogy (CNT), Centre for Energy and Environmental Technologies (CEET), VSB-Technical University of Ostrava, 17. Listopadu 15, 70800 Ostrava, Czech Republic; maria.hasan@vsb.cz (M.H.); szymon.piotr.abrahamczyk@vsb.cz (S.A.); muhammad.aashir.awan@vsb.cz (M.A.A.);; 2 Centre for Nanotechnology (CNT), Centre for Energy and Environmental Technology (CEET), VSB-Technical University of Ostrava, 70800 Ostrava, Czech Republickarla.cech.barabaszova@vsb.cz (K.Č.B.); 3 Faculty of Materials Science and Technology, VSB-Technical University of Ostrava, 17. Listopadu, 70800 Ostrava, Czech Republic; 4 Faculty of Chemistry, Wroclaw University of Science and Technology, Wybrzeże Wyspiańskiego 27, 50-370 Wroclaw, Poland; 5 Key Laboratory of Advanced Carbon Materials and Wearable Energy Technologies of Jiangsu Province, Key Laboratory of Core Technology of High Specific Energy Battery and Key Materials for Petroleum and Chemical Industry, Soochow Institute for Energy and Materials Innovation, College of Energy, Soochow University, Suzhou 215006, China; 6 Institute for Materials Chemistry, IFW Dresden, 20 Helmholtz Strasse, 01069 Dresden, Germany

**Keywords:** CVD-derived graphene quantum dots, carbon nano-onions, graphene nanoribbons, carbon nanowalls, graphene foams

## Abstract

Graphitic nanomaterials have emerged as foundational components in nanoscience owing to their exceptional electrical, mechanical, and chemical properties, which can be tuned by controlling dimensionality and structural order. From zero-dimensional (0D) quantum dots, carbon nano-onions, and nanodiamonds to one-dimensional (1D) nanoribbons, two-dimensional (2D) nanowalls, and three-dimensional (3D) graphene foams, these architectures underpin advancements in catalysis, energy storage, sensing, and electronic technologies. Among various synthesis routes, chemical vapor deposition (CVD) provides unmatched versatility, enabling atomic-level control over carbon supply, substrate interactions, and plasma activation to produce well defined graphitic structures directly on functional supports. This review presents a comprehensive, dimension-resolved overview of CVD-derived graphitic nanomaterials, examining how process parameters such as precursor chemistry, temperature, hydrogen etching, and template design govern nucleation, crystallinity, and morphological evolution across 0D to 3D hierarchies. Comparative analyses of Raman, XPS, and XRD data are integrated to relate structural features with growth mechanisms and functional performance. By connecting mechanistic principles across dimensional scales, this review establishes a unified framework for understanding and optimizing CVD synthesis of graphitic nanostructures. It concludes by outlining a path forward for improving how CVD-grown carbon nanomaterials are made, monitored, and integrated into real devices so these can move from lab-scale experiments to practical, scalable technologies.

## 1. Introduction

Graphitic nanocarbons span a rich hierarchy of morphologies and bonding environments, enabling combinations of high electrical conductivity, large specific surface area, mechanical resilience, and tunable optical/electronic responses that are attractive for catalysis, energy storage, electronics, sensing, and biomedicine [1,2]. In this review, we adopt a dimensional taxonomy to study these materials, as follows: 0D (graphene quantum dots (GQDs), carbon nano-onions (CNOs), and Carbon Nanodiamonds (NDs)), 1D (graphene nanoribbons (GNRs)), 2D (vertically aligned carbon nanowalls (CNWs)), and 3D (interconnected graphene foams (GFs)).

Multiple synthesis routes can access graphitic phases, including arc discharge, laser ablation, electrochemical and liquid-phase methods, hydro/solvothermal chemistry, and template-assisted approaches [3]. These methods have delivered important benchmarks in crystallinity, throughput, and compositional tunability; however, precise morphology control across dimensions and direct device compatibility remain difficult to achieve simultaneously. For example, discharge and ablation excel in crystallinity but have limited scale control; electrochemical exfoliation favors layered products over curved shells; and solution routes offer chemical versatility yet often require post-processing and can struggle with uniform ordering.

Chemical vapor deposition (CVD) stands out for its ability to program nucleation, growth kinetics, and bonding via gas composition, temperature/pressure, substrate interactions, and (when used) plasma or hot-filament activation [4]. Crucially, CVD can form nanographitic structures directly on technologically relevant substrates with controllable dimensionality, from dots, onions, ribbons, and walls to foams, by tuning carbon supersaturation, hydrogen activity, surface energy, and geometric confinement. While CVD typically operates at elevated temperatures and must balance defect generation against growth rate and uniformity, its process tunability and mechanistic transparency make it uniquely suited for comparative, cross-dimensional synthesis.

Despite the extensive literature on individual families (e.g., graphene films or carbon nanotubes), unified perspectives linking CVD growth mechanisms across 0D–3D graphitic architectures remain underdeveloped [5,6].

This review addresses that gap by (i) formalizing the dimensional framework for nanographitic CVD, (ii) comparing how shared control parameters, precursor chemistry, catalyst/support choice, plasma/filament activation, and hydrogen-mediated etching govern nucleation pathways and structural evolution across dimensions, and (iii) highlighting convergent strategies (templating, alloy catalysis, and heteroatom doping) that translate between material classes.

We begin with the fundamentals of CVD activation and catalyst–substrate interactions, then survey synthesis routes and morphology–property relationships across 0D, 1D, 2D, and 3D graphitic systems, including hybrid or hierarchical architectures. The review concludes with comparative spectroscopic insights and an outlook on future directions for scalable, application-oriented CVD synthesis of graphitic nanomaterials.

## 2. Fundamental of Chemical Vapor Deposition

### 2.1. Chemical Vapor Deposition Techniques

CVD relies on the thermal decomposition or catalytic cracking of gaseous precursors on a heated substrate, producing carbonaceous or graphitic films [7,8]. In thermal CVD, hydrocarbons such as methane, acetylene, and ethanol decompose at elevated temperatures, typically between 700 and 1100 °C, over catalytic or bare substrates to yield sp^2^-bonded carbon materials [9]. The relatively high surface diffusion and uniform energy distribution often promote planar film formation, making thermal CVD well suited for graphene layers and carbon nanotubes under appropriate catalyst and temperature conditions. However, depending on gas chemistry, carbon flux, and substrate roughness, fibrous or three-dimensional porous morphologies can also emerge [10].

A related variant, hot-filament CVD (HFCVD), employs a resistively heated tungsten filament operating near 2000–2200 °C to dissociate methane and hydrogen molecules. The resulting atomic hydrogen etches disordered carbon and stabilizes sp^2^- or sp^3^-bonded phases, while the substrate remains cooler (around 950–1000 °C). By adjusting the hydrocarbon-to-hydrogen ratio and total pressure, HFCVD can produce diamond films, graphitic coatings, or fibrous carbon deposits, depending on the local balance between carbon radical generation and hydrogen etching [11,12].

Both thermal and hot-filament methods primarily rely on heat- and radical-driven activation without a deliberately sustained plasma, offering well-characterized chemistry and controllable process windows. These techniques remain fundamental to understand how temperature, carbon supersaturation, and surface diffusion collectively influence the dimensional evolution of graphitic nanostructures.

In contrast to purely thermal approaches, plasma-assisted chemical vapor deposition (PECVD) introduces energetic ions and radicals that activate gaseous precursors at comparatively low substrate temperatures. The presence of a plasma accelerates hydrocarbon dissociation and enhances surface mobility, enabling a wide range of morphologies depending on ion energy, flux direction, and substrate bias. Under higher plasma power or field-aligned conditions, vertically oriented structures such as CNWs or nanofibers are commonly obtained, while moderate ion energies favor conformal or amorphous graphitic films [13].

When the plasma is sustained by microwave radiation (typically 2.45 GHz ≈ 0.122 m), the method is termed microwave-assisted CVD (MACVD). Microwave excitation generates a uniform, high-density plasma at low pressures (1–10 Torr), efficiently dissociating hydrocarbon and hydrogen [14]. The resulting reactive environment supports the controlled synthesis of nanocrystalline diamond, graphitic coatings, or multi-dimensional carbon assemblies, with the final morphology dictated by the hydrogen concentration and plasma uniformity.

Hydrogen plays a dual role in these plasma-based processes: it selectively removes amorphous carbon while stabilizing sp^2^ and sp^3^ bonding configurations, thereby determining whether diamond-like, graphitic, or hybrid carbon phases develop [15,16,17]. The versatility of PECVD and MACVD allows fine-tuning of energy input and gas composition to access diverse carbon dimensionalities, from thin films to vertically aligned frameworks, highlighting their adaptability for both fundamental studies and scalable fabrication.

While activation mechanisms, i.e., thermal, filament-assisted, or plasma-driven, govern how gaseous precursors decompose, the eventual structure and morphology of deposited carbon are strongly influenced by the catalyst and substrate. These parameters determine how activated carbon species nucleate, diffuse, and precipitate to form specific graphitic architectures.

### 2.2. Catalyst and Substrate Effects

Catalysts play a pivotal role in dictating carbon solubility, diffusion, and precipitation behavior during CVD. Transition metals such as Fe, Ni, and Co possess high carbon solubility at elevated temperatures, enabling carbon atoms to dissolve into the catalyst and subsequently precipitate upon supersaturation to form graphitic shells, nanotubes, or nanofibers [18]. The size of catalyst particles determines the density of nucleation sites and ultimately the diameter and morphology of the resulting nanostructures [19]. Equally critical is the catalyst–substrate interface, which governs diffusion dynamics, adhesion strength, and the phase of the precipitated carbon that emerges (as sp^2^ or sp^3^) [20].

Non-reactive template supports such as NaCl [21], MgO [22], and Al_2_O_3_ [23] have been widely employed to minimize interdiffusion between the catalyst and substrate while providing confined nucleation sites. These supports also allow straightforward post-synthesis removal, yielding high-purity carbon nanostructures. For instance, iron nanoparticles supported on NaCl can catalyze the decomposition of acetylene at relatively low temperatures (~400–420 °C), producing CNOs with diameters in the range of 15–50 nm, often encapsulating Fe_3_C cores. Subsequent water dissolution of the salt template results in well-defined, metal-free CNOs [21].

Silicon-based systems illustrate the broader importance of substrate engineering in CVD. At temperatures above ~600 °C, Fe, Ni, and Co react readily with Si to form silicides such as NiSi/NiSi_2_, β-FeSi_2_, and CoSi_x_. This silicidation consumes the active metal, restricts carbon diffusion, and suppresses graphitic nucleation, often yielding amorphous or turbostratic carbon unless a diffusion barrier is introduced [24,25,26]. The strong reactivity of bare silicon further encourages carbide formation, impeding sp^2^-carbon reconstruction. To address these challenges and enable direct CVD growth on silicon, several strategies have proven effective. Introducing ultrathin diffusion or passivation layers, typically SiO_2_, Al_2_O_3_, or TiN films less than 10 nm thick, prevents silicide formation and preserves the catalytic integrity of the metal [27,28,29,30]. Meanwhile, reducing the catalyst thickness to below ~3 nm, commonly achieved through sputtering or e-beam evaporation, minimizes strain mismatch and promotes controlled solid-state dewetting into nanoparticles, resulting in uniform and reproducible nucleation [31]. Surface pre-oxidation of the silicon wafer prior to metal deposition can also be beneficial, as a thin native oxide promotes uniform catalyst dispersion and serves as a mild diffusion barrier. PECVD offers an additional route to achieve graphitic growth at reduced substrate temperatures (≈450–600 °C). In this process, energetic ions and radicals generated by radio-frequency or microwave plasma efficiently dissociate hydrocarbon precursors, promoting vertical growth and controlled heteroatom incorporation without requiring complete carbide breakdown [32]. Recent advances in microwave plasma and surface-wave plasma CVD have further demonstrated that sp^2^-carbon networks can be synthesized at temperatures as low as ~300–500 °C through high-density, low-electron-temperature plasmas [33].

Process optimization also depends on hydrogen control. While the mechanistic role of hydrogen in selective etching and sp^2^ reconstruction has been discussed earlier in the context of plasma CVD, its partial pressure during catalyst-mediated growth critically influences the balance between carbon incorporation and structural ordering. Maintaining a moderate H_2_ concentration helps sustain graphitic nucleation without excessive etching, ensuring uniform film continuity under both thermal and plasma-assisted regimes. When these parameters, that is, barrier layer design, catalyst particle control, plasma activation, and hydrogen balance, are optimally coordinated, direct CVD growth of graphitic layers and nanostructures on silicon becomes feasible, as demonstrated by vertically aligned CNWs and graphene nanosheets growth on Si(100) at 650–700 °C using CH_4_/H_2_ gas mixtures [34].

Beyond catalyst and substrate engineering, feedstock composition can also significantly influence the cleanliness and structural quality of the resulting graphitic films. Jia et al. demonstrated that employing a Cu containing carbon feedstock promotes superclean graphene growth by mediating carbon decomposition and suppressing adventitious contamination during CVD [35].

Having established the fundamental activation mechanisms and examined how catalysts and substrates influence carbon nucleation and growth, it is evident that the underlying process parameters dictate the dimensional evolution of graphitic materials. The combined effects of precursor activation mode, catalyst particle size, substrate interaction energy, and hydrogen concentration determine whether carbon assembles into 0D, 1D, 2D, or 3D frameworks. The subsequent sections discuss these diverse graphitic architectures and their characteristic growth pathways under different CVD environments.

## 3. Graphene Quantum Dots (GQDs)

GQDs are nanoscale particles, typically smaller than 10 nm, whose discrete energy levels arise from quantum confinement and edge or surface states, resulting in size- and structure-dependent optical and electronic properties. In carbon-based systems, the following four major families are recognized: (i) GQDs, which are nanoscale fragments of one or few graphene layers with sp^2^ bonded lattices and tunable edges; (ii) carbon quantum dots (CQDs), which are quasi-spherical, amorphous, or semi-crystalline nanoparticles rich in surface functional groups; (iii) carbon nanodots (CNDs), which consist of largely amorphous carbon cores lacking clear lattice order; and (iv) carbonized polymer dots (CPDs), which are crosslinked polymeric networks incorporating small graphitic domains [36].

Since the synthesis route dictates the structural order, size uniformity, and surface chemistry of GQDs, it directly governs their optical response and potential for device integration. Solution-phase methods, such as hydrothermal, solvothermal, or microwave-assisted, offer scalability and compositional flexibility, and are widely used for producing CQDs, CNDs, and CPDs (with heteroatom doping frequently demonstrated), though their photoluminescence often originates from surface states rather than intrinsic crystal cores [37,38,39].

In general, bottom-up solution-phase routes tend to yield CQDs, CNDs, or CPDs, whereas top-down and vapor-phase techniques are preferred for graphene-derived GQDs. Top-down approaches such as oxidative cutting and electrochemical exfoliation yield GQDs but often suffer from broad size distributions and defect-rich surfaces, leading to excitation-dependent photoluminescence [38]. Template-assisted methods, which confine precursor carbonization within mesoporous or micellar frameworks, achieve improved size control and doping precision but involve multiple processing steps and careful template removal. At the highest level of precision, on-surface molecular assembly under ultra-high vacuum produces atomically defined GQDs, though limited to small scales [40]. While solution-based and top-down strategies dominate in scalability and chemical versatility, the documents only indicate that these methods often produce colloidal, surface-functionalized QDs with PL strongly influenced by surface states, without explicitly stating that ligand stabilization restricts solid–substrate integration [39]. Vapor-phase techniques, in particular CVD, overcome these limitations by directly forming substrate-bound, edge-defined GQDs with minimal surface contamination and enhanced structural precision [37]. Building on this advantage, CVD has emerged as a versatile platform for producing GQDs with controllable dimensions, crystallinity, and edge configuration. Its vapor-phase nature allows direct growth on catalytic and non-catalytic substrates under well-defined thermodynamic conditions, enabling precise control over nucleation and carbon adatom mobility. Unlike solution-based methods that yield ligand-stabilized colloids, CVD growth proceeds in situ on solid surfaces, inherently compatible with device fabrication and integration. These characteristics make CVD an ideal approach for fabricating substrate-bound, high-purity GQDs with tunable morphology and uniform electronic characteristics, setting the foundation for the following discussion on bottom-up CVD routes.

### 3.1. Bottom-Up CVD Routes

Bottom-up CVD synthesis of GQDs provides a controllable route that combines structural precision with direct device integration. This vapor-phase approach enables in situ growth on technologically relevant substrates such as Cu, hexagonal boron nitride (h-BN), and Si [41,42,43], thereby eliminating the need for colloidal processing or capping layers. The resulting GQDs exhibit high crystallinity, clean interfaces, and edge-defined architectures ideally suited for optoelectronic and sensing applications [44,45].

**Figure 1:**
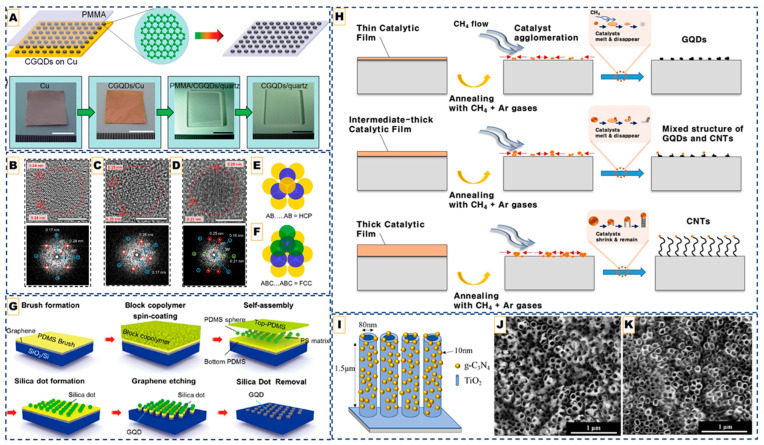
CVD pathways for quantum dot fabrication: Catalyst-mediated CVD approach. (**A**) **Top**: Schematic diagrams of CGQDs formation and post-transfer. **Down**: Corresponding photographs. Scale bar: 1 cm. Reproduced with permission from Ref. [41]. Copyright 2013 WILEY-VCH Verlag GmbH & Co., KGaA, Weinheim. (**B**–**D**) HR-TEM images (**top**) and FFT (**bottom**) of few-layer GQDs. (**E**,**F**) Scheme shows top view of HCP and FCC stacking, respectively, where yellow, blue, and green spheres represent atoms in A, B, and C types of layers, respectively. The scale bar represents 5 nm. Reprinted with permission from Ref. [47]. Copyright 2024 American Chemical Society. (**G**) Top-down CVD-derived quantum dots. Schematic illustration of the fabrication of GQDs including the spin-coating of BCP, formation of silica dots, and etching process by O_2_ plasma. Reprinted with permission from Ref. [48]. Copyright 2012 American Chemical Society. (**H**) Formation mechanism of the GQDs, intermediates, and CNTs in terms of the catalytic-ion dose. (Top to down in the image) Reproduced from Ref. [49]. (**I**) The schematic diagram of TiO_2_-NTA/g-C3N4 (**J**,**K**) Corresponding SEM images of TiO_2_-NTA/g-C_3_N_4_ composites synthesized with 2.0 g and 0.2 g precursor loadings, respectively, showing tunable surface coverage and dot dispersion. Reproduced from Ref. [57].

The formation of GQDs in bottom-up CVD is governed by the competition between carbon adatom nucleation and lateral growth. On catalytic Cu foils, weak carbon–substrate interactions allow for fine control of this balance: at very low methane flow rates, nucleation dominates (N ≫ G), producing dense arrays of nanometer-scale islands that remain spatially confined as GQDs. As illustrated in Figure 1A, this nucleation-dominated regime yields discrete carbon islands that can subsequently be transferred onto flexible supports such as PDMS. When the CH_4_ supply is increased even slightly, lateral growth overtakes nucleation, causing rapid coalescence into continuous graphene films, demonstrating the high sensitivity of CVD growth to gas composition and flow rate [41].

On weakly interacting substrates such as h-BN, adatom mobility is intrinsically limited, leading to spontaneous island formation without the need for catalytic assistance. By tuning CH_4_/H_2_ ratios, the thickness and optical behavior of the dots can be adjusted: monolayer GQDs exhibit broad, red-shifted photoluminescence, whereas multilayer (>10 layers) GQDs display sharper, excitation-independent emission [42]. For more reactive substrates such as Si, rapid nucleation “freezes” adatoms before coarsening, and short dwell times (~1 min at 870 °C) yield ultra-pure one-to-three-layer GQDs whose stable blue emission arises from edge states rather than size confinement [43].

Remote plasma-assisted CVD extends this paradigm by introducing an etch–deposit equilibrium, enabling the growth of ultra-clean ~2 nm GQDs at 580–650 °C. Their sharp density of states and abundant edge sites enhance surface-enhanced Raman scattering (SERS) activity, although plasma exposure can introduce structural defects if not carefully managed [44]. Moreover, a catalyst-free variant of plasma-assisted CVD further demonstrated that graphene nanoislands grown directly on Si/SiO_2_ can exhibit intrinsic quantum-dot behavior. Using microwave plasma CVD below 400 °C [46], 30–70 nm graphene domains that displayed clear Coulomb-blockade features and twofold degeneracy in low-temperature transport were obtained, confirming electronic quantization within CVD-grown islands. This work underscores that bottom-up vapor-phase routes can not only yield atomically defined GQDs, but also integrate quantum functionality directly onto semiconductor platforms.

In summary, bottom-up CVD approaches leverage the intrinsic thermodynamics of carbon nucleation on surfaces like Cu and h-BN, offering compatibility with standard CVD infrastructure and scalability to wafer-level substrates. Their ability to tune dot density, thickness, and emission through simple parameters, that is, gas flow, substrate type, and dwell time, makes them more practical than multi-step synthetic routes. Nonetheless, maintaining size uniformity and reproducibility across large areas remains a key challenge for translating CVD-grown GQDs into reliable device platforms.

### 3.2. Top-Down CVD-Derived Approaches

Beyond direct nucleation, alternative fabrication strategies have been developed where pre-formed graphene serves as the starting material. In this approach, pre-formed graphene films are selectively cleaved into nanoscale domains rather than being nucleated from gaseous precursors. For instance, electrochemical cutting of CVD graphene oxidatively “unzips” the lattice along defect lines, yielding 3–8 nm GQDs that preserve multilayer AB/ABC stacking order (Figure 1B–F). This retained stacking sequence is uncommon in nucleated dots and enhances interlayer coupling, making such GQDs promising for correlated-electron and quantum transport studies [47].

Alternatively, nanoscale patterning can be achieved by block copolymer self-assembly. Here, polymer micelles form ordered silica nanodot masks on the graphene surface, enabling oxygen plasma etching to sculpt uniform 10–20 nm GQDs. In this method, mask geometry defines dot size and pitch, whereas plasma dose controls edge oxygenation and photoluminescence intensity (Figure 1G) [48].

Although these top-down conversion routes provide exceptional precision in size and edge chemistry, these remain process-intensive and low-throughput, limiting their applicability for wafer-scale synthesis. Nevertheless, their atomically defined edges and tunable confinement make them invaluable research tools for probing quantum confinement, spin coupling, and correlated-electron effects where structural precision outweighs scalability.

### 3.3. Catalyst-Mediated CVD Strategies

While top-down CVD routes rely on physical or chemical fragmentation, introducing catalytic nanoparticles offers an additional degree of spatial and compositional control. Here, metal nanoparticles are often introduced as transient catalytic seeds to localize nucleation or tune dimensionality. Fe implantation into Si produces in situ Fe nanoparticles that nucleate GQDs; during high-temperature growth, these particles diffuse, leaving patterned, metal-free arrays [45]. While effective for deterministic positioning, this route introduces additional complexity and possible contamination. Pt sputtering provides another perspective: the size of Pt nanoparticles determines the carbon phase. Small, unstable droplets catalyze GQDs, whereas larger, stable particles persist to drive CNT growth, as shown in Figure 1H [49]. This highlights the ability of nanoparticle systems to map 0D–1D phase boundaries, but at the cost of extra processing steps and the risk of residual metal. Catalyst-mediated methods are less commonly adopted for applications because residual metals compromise purity and removal steps introduce complexity. However, these are still studied because these provide mechanistic insight into carbon phase evolution and enable site-selective nucleation, which is valuable for device integration where deterministic placement of QDs is needed.

### 3.4. Doped and Hybrid Systems

Expanding beyond morphology control, doping and hybridization strategies allow compositional and electronic tuning during CVD growth. In twisted bilayer graphene grown by CVD and supported on SiO_2_/Si, drop-cast CQDs exhibit twist-angle-dependent photophysics. When the twist angle (θ) is small (θ ≲ 11°), photoluminescence (PL) is enhanced due to the suppression of non-radiative charge transfer, making the CQDs more photoluminescent. However, at θ ≈ 0°, where the graphene layers are AB-stacked, and in single-layer graphene, PL is strongly quenched. As the twist angle increases (θ ≳ 15°), the bilayer graphene approaches SL-like behavior, and PL becomes weaker than in the small-angle regions. This behavior can be attributed to a competition between radiative and non-radiative electron-transfer channels, which is modulated by interlayer coupling and Fermi-velocity renormalization [50].

In addition to this observation, a novel mechanism for CQD formation has been demonstrated using ethanol on eggshell-derived Fe-Co/CaO catalysts. At approximately 850 °C with a 30 min dwell time, ethanol-derived OH-radicals are proposed to oxidatively corrode carbon nanofibers (CNFs) into ~2–3 nm CQDs, which then anchor to carbon nanotube (CNT) walls. Interestingly, the process is time- and temperature-dependent: shorter growth times (≈10 min) favor the formation of CNF/CNT hybrids, whereas longer growth times (≈60 min) or higher temperatures lead to the over-oxidation and consumption of CQDs, ultimately leaving only CNTs behind [51]. This observation reveals that CQDs can not only form by direct nucleation but also by secondary transformation of existing carbon nanostructures during the growth process, although their stability window remains narrow. These findings are important for developing new optoelectronic and sensing platforms, where controllable CQD formation is crucial.

Beyond structural integration, CVD also allows for atomic-level compositional tuning, such as the incorporation of nitrogen into GQDs (N-GQDs). The specific nitrogen doping configuration, whether pyridinic, pyrrolic, or graphitic, depends on the precursor chemistry and growth temperature. Pyridinic and pyrrolic nitrogen configurations introduce mid-gap states, which red-shift emission and enhance charge-transfer efficiency, while graphitic nitrogen improves conductivity and stabilizes edge states [52,53,54]. This capacity to precisely program dopant configurations in CVD showcases how process parameters can influence electronic structure in carbon materials.

While both N-GQDs and graphitic carbon nitride quantum dots (g-CNQDs) contain nitrogen, these are fundamentally different material classes. N-GQDs retain the sp^2^-bonded carbon lattice of graphene, with nitrogen atoms incorporated as dopants in graphitic configurations. These dopants locally modify the electronic structure, tuning photoluminescence and charge-transfer properties. In contrast, g-CNQDs feature an intrinsic sp^2^-hybridized C–N network derived from graphitic carbon nitride (g-C_3_N_4_), where nitrogen forms part of the polymeric backbone rather than acting as a dopant. The optical and electronic behavior of g-CNQDs arises from π–π* and n–π* transitions within triazine or heptazine units, which lead to strong visible-light absorption and pronounced photocatalytic activity [55,56].

Despite both classes containing nitrogen, the differences between N-GQDs and g-CNQDs lie in their structural origins and the role of nitrogen. N-GQDs are synthesized through CVD or plasma-assisted routes by incorporating nitrogen into graphene. In contrast, g-CNQDs are formed via the thermal condensation of nitrogen-rich precursors, such as melamine or dicyandiamide, and cannot be synthesized through conventional hydrocarbon CVD. Recent low-pressure two-zone vapor-phase CVD routes have demonstrated the formation of g-C_3_N_4_ nanoparticles through confined sublimation–polymerization of melamine vapors at ~750 °C (region 1) onto TiO_2_ nanotube arrays held at ~500 °C (region 2) under a 40–133 Pa Ar atmosphere. This process enables the formation of uniform, sub-10 nm g-C_3_N_4_ nanoparticles that are anchored within the nanotube channels, preventing gas-phase aggregation and over-polymerization and offering a scalable route for photocatalytic applications, as shown in Figure 1I–K [57].

Such fine control over melamine polymerization at 750 °C (region 1) and 500 °C (region 2) suppressed defect formation and aggregation, allowing g-C_3_N_4_ to deposit as discrete nanoparticles rather than films. The resulting TiO_2_-NTA/g-C_3_N_4_ heterostructure formed efficient Z-scheme junctions, promoting rapid charge separation, and markedly enhanced photocatalytic hydrogen evolution compared to pristine TiO_2_ or bulk g-C_3_N_4_. This work underscores how CVD process engineering, particularly reduced-pressure polymerization and nanoscale confinement, can be leveraged to achieve intimate semiconductor coupling and defect-controlled carbon nitride growth, a strategy extendable to other hybrid quantum or 2D architectures.

While GQDs represent 0D manifestations of sp^2^ carbon, their structural evolution under continued graphitization leads naturally to multi-shell architectures, the CNOs discussed next.

## 4. Carbon Nano-Onions (CNOs)

CNOs, also known as onion-like carbons (OLCs), are multi-shell graphitic nanostructures composed of concentric sp^2^ carbon layers that resemble nested fullerenes. These are typically categorized based on their core structure into the following three main classes: solid (filled) onions, hollow onions, and core–shell architectures. The core–shell structures contain either a metallic nanoparticle or a void enclosed by the carbon shells [58,59]. While some authors use the term “yolk–shell” for the latter case, core–shell more accurately conveys the presence of a distinct core enclosed by graphitic shells. These variants share similar sp^2^ bonding but differ in internal morphology and, consequently, in their electronic and mechanical properties.

Several established methods yield CNOs, each with advantages and constraints. Arc discharge forms highly crystalline onions via plasma condensation but affords limited size control and scale [60]. Thermal conversion of nanodiamond at high temperatures produces well-ordered 3–10 nm onions, yet requires extreme heat and can induce coalescence. Flame pyrolysis from carbon-rich fuels (e.g., ghee, candle wax, and plant or waste oils) offers simple, low-cost, catalyst-free production of ~20–40 nm onions with a high surface area, though morphology depends strongly on flame conditions and collection substrates. Pyrolysis of molecular precursors such as hexachlorobenzene, sodium azide, or ferrocene can yield larger hollow onions (~50–100 nm) but often shows variable shell number and uniformity. Additional routes, including ion implantation, laser ablation, and microwave and electrochemical syntheses, can generate CNOs or functionalized variants, yet reproducibility, yield, and tight control over shell count remain recurring challenges. These constraints collectively motivate exploration of vapor-phase strategies for finer morphology control and improved scalability [60,61].

CVD offers fine control over carbon supply, temperature, and the local catalyst or template environment. It enables single-step synthesis of hollow, filled, or core–shell onions at moderate temperatures from hydrocarbon feeds. The method scales to large substrates and templates and balances gas-phase decomposition, surface diffusion, and precipitation to drive the transition from amorphous to graphitic multi-shell structures [60].

### 4.1. CVD Growth Mechanisms of CNOs

The formation of carbon onions and related graphitic shells by CVD is strongly influenced by the choice of hydrocarbon precursor, growth temperature, and especially the catalyst or template employed. CVD provides a controllable route where carbon is released from the precursor and reorganizes into multilayer sp^2^ shells under conditions defined by the substrate or nanoparticle surface. The resulting structure, metal-filled onions, hollow onions, or hollow graphitic shells, arises primarily from catalyst/template chemistry rather than from a universal kinetic model. For example, ethylene CVD over Co/MgO at 700 °C produces metal-encapsulated carbon onions that become hollow after acid removal [62], while methane CVD at 600 °C over Ni/Al yields both Ni-filled and hollow onions directly depending on the Ni particle state during growth [63]. Likewise, ethanol CVD at 775 °C coats oxide nanoparticles with few-layer graphene, and etching the oxide templates produces hollow graphitic shells shaped by the underlying particle morphology [64]. Across these systems, the catalyst or template determines whether carbon precipitates as filled, hollow, or template–replica shells, whereas precursor and temperature mainly affect the degree of graphitization and layer uniformity. These experimentally observed morphologies are further consistent with atomic-scale simulations, where DFT studies show that five-member carbon rings form more readily than six-member rings during carbon condensation. Such pentagonal loops introduce local curvature and act as intrinsic nucleation sites for closed graphitic shells, providing a microscopic basis for the curvature-driven closure observed during CVD growth and reinforcing the role of catalyst-induced surface conditions in selecting between filled and hollow onion structures [65].

Overall, the combination of core type (solid, hollow, and core–shell) and shell characteristics (thickness, defects, and dopants) is set by the synthesis strategy. Table 1 summarizes representative CVD-derived hollow and core–shell carbon nanostructures, highlighting the influence of catalyst/template type, feedstock, and growth temperature on resulting morphology and mechanism.

### 4.2. Metal-Catalyzed CVD Systems

In transition-metal-assisted CVD, metals such as Ni, Fe, and Si mediate carbon uptake and precipitation through a classical dissolution–diffusion–precipitation cycle [75]. Hydrocarbon molecules dissociate on the metal surface, and carbon atoms dissolve into the subsurface lattice. When supersaturation occurs, carbon precipitates outward, forming successive sp^2^ shells that encapsulate the catalyst particle. This process of solid-state graphitization under carbon supersaturation is fundamental to metal-driven CVD.

Nickel remains the archetypal catalyst due to its optimal balance of carbon solubility and catalytic activity. In Ni/Al_2_O_3_ systems [63], CVD growth at 600 °C enables smooth formation of hollow graphitic onions (<50 nm) through a Kirkendall effect, where outward carbon diffusion and partial metal contraction during cooling create a central void. The Al_2_O_3_ support prevents Ni sintering and maintains nanoparticle dispersion, ensuring uniform onion formation.

Alloying further refines this behavior. In Ni-Fe catalysts [75], methane decomposition between 750 and 950 °C forms a transient Fe-Ni-C austenitic phase with high carbon solubility. Upon cooling, carbon precipitates to yield multi-shell onions or core–shell particles depending on composition. Nickel accelerates hydrocarbon cracking [75,76], while iron enhances crystalline ordering, thus achieving polyhedral, hollow onions with 10–20 graphitic layers. At excessive carbon flux, carbide-encapsulated particles form, which can later be purified by acid etching.

Cobalt-based systems (e.g., Co/MgO) [62] demonstrate comparable control, with MgO acting as a dispersant and anti-sintering support. Adjusting Co content between 1 and 10 wt% tunes particle size and shell uniformity, producing hollow onions (10–50 nm) after removal of the oxide and metal residues.

**Figure 2:**
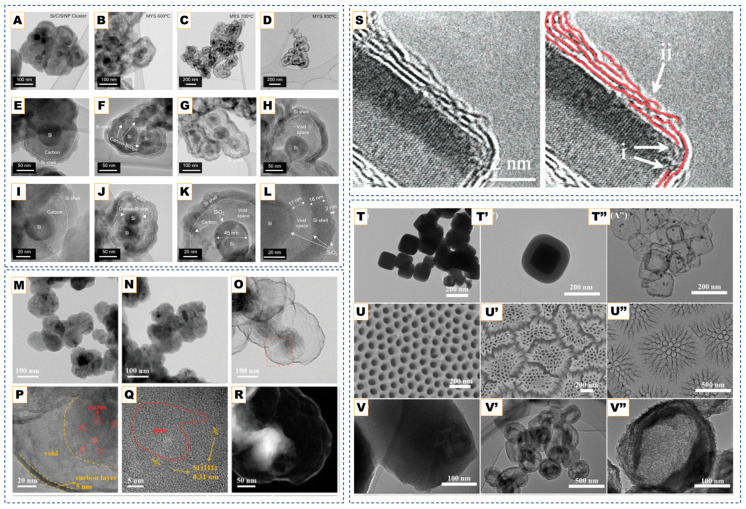
(**A**–**L**). SEM images of graphitized hollow carbon spheres and yolk-structured carbon spheres fabricated by metal-catalyst-free chemical vapor deposition. Adapted from Ref. [77]. TEM images of (**M**) Si, (**N**) Si@C, and (**O**,**P**) pSi@void@NFC composites. (**Q**) HRTEM image of pSi@void@NFC composites. (**R**) HAADF-STEM image, and EDX mapping of Si, O, C, F, and N elements of pSi@void@NFC composites. TEM images of the hollow carbon onions obtained from various cobalt nitrate and basic magnesium carbonate ratios. Reprinted with permission from Ref. [71]. Copyright 2024 Wiley-VCH GmbH. (**S**) TEM images of a MgO crystal coated in graphene layers. Graphene layers are highlighted in red shows (i) Graphene layer roots on the MgO crystal. (ii) Wrinkle formation due to growth process. Reprinted with permission from Ref. [79]. Copyright 2007 American Chemical Society. (**T**,**T′**,**T″**) TEM images of cubic Cu_2_O@SiO_2_, and the final yolk–shell structured Cu@C after templates removal. (**U**,**U′**,**U″**) SEM images of AAO template and the resultant carbon nanotubes arrays. (**V**,**V′**,**V″**) TEM images of the mesoporous SiO_2_ particles and the porous carbon particles. Reproduced with permission from Ref. [68]. Copyright 2020 John Wiley & Sons.

Although not metal-catalyzed, a recent study by Kim et al. [77] introduced a multilayered Core–Shell (MYS) framework featuring a carbon-bridge connection between the core and outer graphitic shells, offering complementary insights into concentric carbon-shell design relevant to CNO engineering. Their CVD-assisted synthesis employed sequential silane and acetylene deposition followed by controlled carbon oxidation, producing a Si@C@SiOx/Si/SiOx architecture in which the carbon bridge preserved electronic continuity while the void space buffered mechanical stress. This hierarchical design effectively mitigated interfacial isolation, a problem analogous to electron-transport loss in hollow CNOs, by enabling simultaneous accommodation of core expansion and retention of high conductivity. The MYS structure exhibited a high reversible capacity (2802 mAh g^−1^) and remarkable structural stability over repeated cycling, underscoring how bridged, multi-shell configurations can unify the advantages of hollow and core–shell carbons. Such strategies highlight the broader potential of CVD processes to create electronically coupled concentric carbon frameworks with tunable interfaces for energy-storage and catalysis applications. The structural evolution of the multilayered core–shell framework under different annealing conditions further demonstrates how thermal treatment governs carbon ordering and shell densification. This progression is illustrated below in Figure 2A–L.

These studies collectively highlight how catalyst composition, phase, and dispersion govern the precision achievable in CVD-grown graphitic shells. Under moderate temperatures (∼750–850 °C) and controlled carbon flux, transition-metal catalysts such as Ni, Fe, or Ni-Fe guide carbon dissolution and re-precipitation to yield multi-shell or polyhedral carbon onions whose graphitic thickness ranges from only a few layers up to several tens, depending on the catalyst and reaction conditions. Increasing temperature or employing catalyst phases with higher carbon solubility produces shells with higher graphitic crystallinity (lower ID/IG), indicating that the resulting morphology reflects the balance between carbon uptake, diffusion, and precipitation at the catalyst interface rather than a fixed layer-by-layer growth regime. When oxide templates or multi-component interfaces are introduced, similar CVD principles extend naturally to the formation of core–shell, hollow, or hierarchical carbon architectures, demonstrating the broad applicability of catalyst- and interface-mediated graphitic structuring across carbon-onion and related framework materials [76,77]. For instance, in hybrid systems such as Au@hmC-FeCo [59], Fe-Co nanoparticles catalyze local graphitization while the Au core preserves a central void, creating magnetically active core–shell composites. Similarly, SiO_2_ or Au@SiO_2_ spheres serve as inert templates, directing methane-based CVD growth to produce hollow or core–shell structures after etching. These designs illustrate how the controlled coupling of diffusion, catalysis, and templating can convert simple concentric onions into mechanically robust, electrically continuous architectures suitable for energy storage and catalytic applications, as can be seen in Figure 2M–R [69,70,71,78].

### 4.3. Oxide-Templated CVD

Beyond metal catalysis, oxide-templated CVD exploits geometric confinement rather than catalytic activity to organize graphitic shells, as developed by Rümmeli et al. [79]. Oxides such as Al_2_O_3_, MgO, and SiO_2_ provide high-energy surfaces that promote carbon rearrangement during hydrocarbon pyrolysis, allowing curved sp^2^ layers to replicate the template morphology, as shown in Figure 2S. Ethanol-based CVD over MgO, Al_2_O_3_, or TiO_2_ nanoparticles produces few-layer carbon shells with 3–7 graphitic layers, which can be functionalized with -OH or -SH groups following mild oxidation [64]. Similarly, SiO_2_ or Au@SiO_2_ templates yield uniform hollow or Au@graphene core–shell hybrids after HF etching [67]. In these systems, the template dictates curvature and shell integrity, while inert cores (e.g., Au) preserve structure during growth.

Recent refinements [66] achieve highly graphitized hollow spheres with exceptional uniformity and electrochemical durability. The oxide simultaneously serves as a mold and graphitization promoter, stabilizing curved carbon at the interface. Variants of non-isothermal CVD employ polyphenol vapor and SiO_2_-based templates, as shown in Figure 2T–V [68]. These further demonstrate tunable thickness and shell cohesion via temperature gradients. These approaches confirm that oxide templating provides a robust, metal-free pathway to carbon shells with high purity, uniformity, and chemical functionality.

### 4.4. Heteroatom-Doped and Functionalized Carbon Hollow Shells

Heteroatom doping marks a pivotal evolution in CVD-based carbon nanostructure design, transforming inert graphitic frameworks into electronically and chemically tunable architectures. Pristine carbon shells exhibit high conductivity and mechanical resilience but limited surface reactivity. The introduction of dopants such as B, N, P, or Al breaks this symmetry, introducing lattice strain, charge polarization, and active sites for catalysis and ion transport.

Each dopant contributes distinct electronic effects. Boron, being electron-deficient, induces p-type behavior and enhances oxidative catalytic activity. In boron-assisted CVD, co-feeding boron precursors with hydrocarbons yields substitutional incorporation within the sp^2^ lattice, stabilizing graphitic order while enriching π-electron localization [80,81]. Nitrogen, introduced through NH_3_-assisted CVD, donates electrons to the π-system, creating pyridinic, pyrrolic, and graphitic configurations that increase interlayer spacing, wettability, and active site density, benefiting both energy storage and catalytic processes [74]. Phosphorus doping via triphenylphosphine vapor introduces lattice expansion and localized charge variations that facilitate ion intercalation and redox reactions, improving electrode performance [73]. Co-doping strategies amplify these effects. Yu et al. [72], reported Al-B-N co-doped core–shell carbons produced by CVD, where electron-deficient (Al and B) and electron-rich (N) dopants cooperatively modulate charge distribution and defect stabilization. The resulting hierarchical graphitic frameworks exhibited enhanced conductivity, enlarged ion-accessible surface area, and superior cycling stability, demonstrating the synergistic potential of multi-element doping in tuning the structural and electronic landscape of CVD-derived carbon nanostructures.

The controlled assembly of sp^2^-bonded carbon shells demonstrates how CVD can tailor concentric architectures by manipulating diffusion, catalysis, and templating. Yet, CVD’s versatility extends further: by adjusting gas composition, temperature, and plasma parameters, it can stabilize sp^3^-bonded carbon and produce NDs with equally rich dimensional and defect control. To underscore this broader capability of vapor-phase synthesis, the following section examines the mechanisms and parameters that govern NDs formation, contrasting the sp^2^-based onion structures with sp^3^-dominated NDs frameworks.

## 5. Carbon Nanodiamonds (NDs)

The synthesis of NDs through CVD represents one of the most versatile manifestations of carbon chemistry under non-equilibrium conditions. Unlike detonation or high-pressure high-temperature (HPHT) processes, which rely on explosive quenching or pressure–temperature regimes analogous to Earth’s mantle, CVD permits atomistic control of carbon bonding pathways through the independent regulation of radical flux, hydrogen activity, and substrate energetics. This decoupling of thermodynamic and kinetic variables allows for the stabilization of sp^3^-bonded carbon far from equilibrium via hydrogen-mediated surface reactions, giving rise to diverse ND forms ranging from discrete particles to continuous ultra-nanocrystalline films [82,83,84].

**Figure 3:**
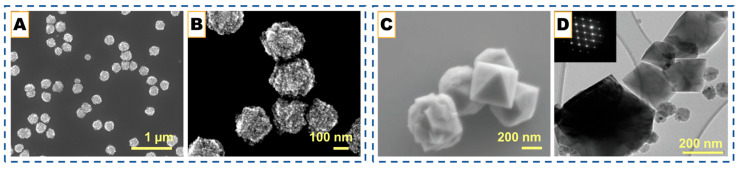
Morphological signatures of NDs’ formation under distinct CVD regimes. (**A**,**B**) Gas-phase nucleation through pinhole-assisted collection, confirming particle condensation independent of the substrate. Reproduced from Ref. [84]. (**C**,**D**) Seeded-film growth on Si(100) demonstrating selective seed survival and crystalline faceting; inset diffraction pattern confirms sp^3^ order. Reproduced from Ref. [88]. Copyright 2019 WILEY-VCH Verlag GmbH & Co. KGaA, Weinheim.

NDs generated by CVD can be broadly grouped into several categories distinguished by their structural origin and synthesis environment. The first comprises loose, substrate-free ND particles that nucleate directly in the gas phase of CH_4_/H_2_ plasmas or hydrocarbon flames. Studies have reported evidence of gas-phase NDs formation even when the substrate was physically isolated from the plasma, suggesting that homogeneous nucleation can occur under sufficiently high carbon supersaturation and hydrogen radical flux (Figure 3A,B) [84]. Nitrogen addition during MPCVD consistently enhances secondary nucleation, leading to dense, ultrafine grains characteristic of ultra-nanocrystalline diamond films. In these environments, increasing nitrogen flow modifies plasma chemistry, most notably by strengthening C_2_ and CH radical emission, and drives repeated renucleation that reduces grain size to only a few nanometers. A further category within CVD-grown nanodiamond materials includes films whose microstructure and properties are deliberately tuned through controlled incorporation of nitrogen or other additives, which alter grain boundary chemistry, sp^2^/sp^3^ ratios, and defect populations, as demonstrated in the nitrogen-doped UNCD films discussed in these studies [85,86,87]. Increasing C_2_ concentration or nitrogen doping promotes frequent re-nucleation, yielding ultrafine grains consistent with UNCD textures. A third category encompasses doped NDs intentionally engineered to host color centers such as NV, SiV, and GeV during or after growth. These are typically formed under controlled nitrogen or Group-IV precursor additions in microwave plasma environments optimized for defect incorporation, as shown in Figure 3C,D [88,89]. Finally, there are seeded-film NDs, nuclei derived from detonation, or HPHT powders that survive the initial etching phase and serve as crystallization sites for further growth. Their stability depends on local hydrogen and oxygen concentrations, which determine whether small seeds (<4 nm) are etched away or stabilized for coalescence [90,91].

Although non-CVD methods such as detonation, HPHT, and laser ablation are indispensable for bulk production, these inherently lack the kinetic precision that defines CVD. Detonation NDs, formed by the adiabatic expansion of carbon-rich explosives, contain extensive sp^2^ shells together with metal impurities, and, therefore, require aggressive purification using strong oxidizing acids to remove graphitic shells and contaminants [92,93]. HPHT methods produce highly crystalline but micrometric diamonds unsuitable for nanoscale control, while laser and arc techniques generate amorphous mixtures with uncontrolled defect chemistry. In contrast, CVD offers a continuous tuning capability between amorphous carbon, nanographite, NDs, and single-crystal diamond, all within a unified plasma–chemical framework [82,83].

### CVD Routes and Control Parameters

Among CVD modalities, microwave plasma chemical vapor deposition (MPCVD) remains a leading approach for ND and UNCD-like film synthesis because it provides controllable plasma environments and stable hydrogen chemistry while allowing precise adjustment of CH_4_/H_2_ ratios and power inputs [83,85]. Under nitrogen addition, or more generally, under conditions that increase the intensity of C_2_ and CH radicals, secondary nucleation is strongly promoted, yielding ultrafine grains and dense grain-boundary networks characteristic of UNCD structures [85,86,87]. Hot-filament CVD, although technologically simpler, is limited by filament heating and thermal loading, yet remains useful for low-cost diamond and ND coatings when filament temperature and spacing are properly controlled [93]. Alternative plasma geometries, including linear-antenna or vertical-substrate MPCVD arrangements, extend diamond growth to larger areas or exploit spatial gradients in hydrogen and temperature to reveal systematic variations in nucleation and grain size across a single wafer [94].

Oxygen plays a decisive role during early growth: at sufficiently low concentrations, it can suppress sp^2^ phases, whereas concentrations approaching or exceeding ~1 vol% under typical MPCVD pressures rapidly etch ND seeds and suppress nucleation unless a pre-grown diamond layer is present [91]. Seed survival is especially sensitive during the incubation stage, where small detonation NDs (<4 nm) can be removed by hydrogen-driven etching before contributing to film coalescence [90]. Substrate seeding density and nucleation enhancement strategies, therefore, remain critical; seed densities at or above ~10^11^ cm^−2^ are typically required for the formation of continuous ND films [82,90].

Nitrogen constitutes another key parameter, modifying plasma chemistry and grain-boundary structure. Sub-percent additions enhance secondary nucleation and smooth film morphology, whereas higher nitrogen concentrations increase sp^2^-rich grain boundaries and degrade structural and mechanical uniformity [86,87]. Inert gases such as argon or helium also influence plasma chemistry; argon dilution, in particular, increases C_2_ emission and can facilitate ultrafine grain formation [85,95], while helium dilution reduces C_2_ to below detectable levels without preventing nanocrystalline growth [96].

Across all CVD processes, the fundamental growth mechanism remains based on CH_3_ incorporation at hydrogen-terminated sp^3^ sites, with atomic hydrogen continually abstracting and restoring surface hydrogen to generate active sites for further carbon addition [82,97,98]. Although the relative contributions of CH_3_-based versus C_2_-assisted pathways depend on gas mixture and plasma conditions, the CH_3_-H mechanism remains consistently supported for microcrystalline and nanocrystalline regimes across the systems examined.

The major limitations identified in the literature stem from hydrogen-mediated etching during incubation, renucleation-driven grain boundary formation under high reactive-carbon or nitrogen flux, and process sensitivity to oxygen concentration, which are factors that can delay coalescence or increase sp^2^ content if not carefully managed [83,87,90].

Improvement strategies focus on kinetic control rather than equipment redesign. Programmed incubation sequences, in which the CH_4_ fraction is briefly elevated during seeding to protect small nuclei and then reduced once coalescence begins, have proven effective in stabilizing sub-4 nm seeds [90]. Controlled trace oxygen dosing (<2%) suppresses sp^2^ formation while minimizing oxidative seed loss, particularly in low-temperature MPCVD on glass substrates [91,99].

Nitrogen can be harnessed as a deliberate promoter of re-nucleation if maintained below threshold levels that induce graphitic boundary growth [86,87]. Argon-assisted regimes exploit C_2_ chemistry for ultrafine grain refinement while retaining sufficient hydrogen selectivity [85,100]. Reactor designs incorporating built-in hydrogen/temperature gradients accelerate optimization, while OES feedback enables real-time control of the CH_3_/H ratio in the ND growth window [85,94].

In parallel, advances in dopant control and color-center engineering demonstrate how ND synthesis by CVD can be integrated with quantum materials design. Co-feeding nitrogen or Group-IV precursors allows for in situ formation of NV, SiV, or GeV centers without post-growth implantation, provided that plasma chemistry balances defect incorporation with grain-boundary suppression [88,89]. In summary, CVD offers a uniquely tunable platform for controlled ND synthesis, enabling manipulation of sp^3^ nucleation and growth pathways through coordinated control of CH_3_ flux, hydrogen concentration, dopant chemistry, and plasma geometry. The mechanistic framework, anchored in the interplay between deposition and hydrogen-mediated etching, has matured to the point that ND synthesis can now be regarded as a continuum rather than a discrete technique. The path forward lies in mastering plasma–surface interactions to balance re-nucleation, seed survival, and impurity control with atomistic precision, while continuing to refine plasma modulation and scalable reactor architectures.

Having explored how CVD can be tuned to stabilize sp^3^-bonded carbon in the form of NDs, we now turn to its ability to engineer one-dimensional sp^2^-bonded structures. GNRs occupy an intermediate dimensionality between zero-dimensional dots and two-dimensional sheets; these combine high carrier mobility with tunable bandgaps defined by their width and edge configuration. The next section surveys CVD routes to GNRs, highlighting how substrate anisotropy, kinetic control, and template confinement transform inherently two-dimensional graphene growth into ribbon-like architectures.

## 6. Graphene Nanoribbons (GNRs)

GNRs combine the high carrier mobility of graphene with tunable, width-dependent bandgaps, making them promising candidates for next-generation logic, interconnect, and quantum devices [7]. Early studies of graphene CVD on germanium established that substrate crystallography governs both domain symmetry and growth anisotropy. On Ge(110), graphene develops elongated, anisotropic domains due to differences in armchair and zigzag edge propagation, while Ge(100) produces more symmetric, often hexagonal grains [101]. Subsequent work comparing thermal CVD and PECVD growth on Ge surfaces showed that graphene morphology, strain state, and grain elongation depend strongly on the underlying orientation: Ge(001) and Ge(110) promote elongated domains and directional growth, whereas Ge(100) tends toward more compact grains. PECVD further enabled lower-temperature growth and revealed systematic tensile or compressive strain signatures depending on the method, reinforcing the critical role of Ge orientation in determining graphene shape, strain, and growth kinetics [102].

In parallel, independent thermal annealing studies revealed how post-growth high-temperature treatments transform ribbon edges and lattice structure. Annealing up to 2800 °C progressively heals point defects, straightens lattice fringes, and converts reactive open ribbon edges into single-, double-, and multi-loop closures, yielding more graphitized and structurally ordered nanoribbons with reduced Raman D-band intensity [103]. These insights clarified how GNR edges stabilize under extreme thermal processing.

Ge(001) CVD experiments then demonstrated that narrow armchair-edge graphene nanoribbons can form spontaneously during methane CVD, exhibiting smooth edges and semiconducting transport behavior, although unseeded growth typically yields two degenerate ⟨110⟩ orientations and substantial width variability [104,105]. Building directly on crystallographic and strain insights from earlier CVD studies, this work positioned Ge(001) as a platform for inherently anisotropic ribbon formation.

Further refinement arrived with seeded CVD approaches. Polycyclic aromatic hydrocarbons such as PTCDA and pentacene enable quasi-synchronous nucleation, suppress spontaneous methane nucleation, and yield narrower, more uniform ribbons. Under ~920 °C and ~0.5–0.9% CH_4_, seeded ribbons reach ~3–4 nm widths with polydispersities of 11–15%, and exhibit strongly anisotropic growth where length increases far more rapidly than width [106].

Orientation control was subsequently achieved using vicinal (miscut) Ge(001) substrates, which break the symmetry between the two ⟨110⟩ directions. Under slow growth, more than 90% of ribbons align unidirectionally, and sub-10 nm semiconducting ribbons incorporated into FETs reach on/off ratios of ~570 and conductance values of ~8 µS [107].

Kinetic Monte Carlo modeling later reproduced these experimental regimes and attributed high-aspect-ratio ribbon formation to direction-dependent stabilization and conversion of edge-bound precursor species. At low precursor densities, which correspond to slower growth, ribbons tend to extend mainly along their length, while higher precursor densities encourage more lateral growth, and, therefore, produce ribbons with lower aspect ratios [108]. Beyond chemically driven anisotropy, template- and etching-guided strategies introduce physical confinement as a means to direct nanoribbon growth. In these approaches, directional control is decoupled from substrate chemistry. Chen et al. demonstrated CVD growth inside pre-etched monolayer-deep h-BN trenches, typically narrower than 10 nm, whose zigzag-oriented walls constrain the GNR edges and enforce crystallographic alignment. The ribbons grow embedded within the dielectric host, eliminating any need for post-growth transfer, and AFM measurements confirm atomically smooth, epitaxial interfaces. Room-temperature transistors fabricated from sub-10 nm embedded ribbons exhibit on/off ratios exceeding 10^4^ [109].

In contrast, Cai et al. realized a lithography-free, template-free method on liquid Cu substrates, where hydrogen-regulated, comb-like surface etching spontaneously forms parallel channels that guide ribbon growth. This process yields monolayer GNRs with smooth edges, micrometer-scale lengths (≈3–5 μm), and controllable widths, including sub-10 nm ribbons, all aligned through self-assembly on the liquid metal surface [110].

**Figure 4:**
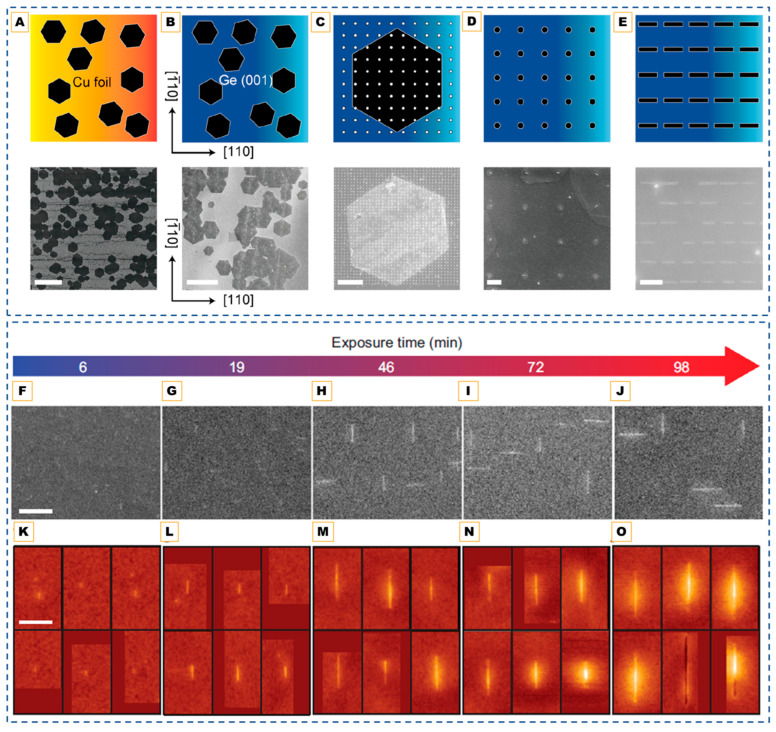
(**A**) Hexagonal crystals of monolayer graphene are grown on Cu foil. (**B**) The hexagonal graphene crystals are transferred onto Ge(001). (**C**) Arrays of Al dots are patterned on top of the hexagonal graphene crystals on Ge(001) via electron-beam lithography, development, Al deposition, and lift-off. (**D**) The exposed graphene that is not protected by the Al masks is etched using an oxygen reactive ion plasma and then the Al masks are etched with H_3_PO_4_, resulting in an array of circular graphene seeds on Ge(001). (**E**) Graphene is grown from the seed array via CVD. Scale bars are 80 μm (**A**), 40 μm (**B**), 4 μm (**C**), and 200 nm (**D**,**E**). Reprinted with permission from Ref. [111]. Copyright 2018 American Chemical Society. (**F**–**J**) SEM images of nanoribbons after CH_4_ exposure times of 6, 19, 46, 72, and 98 min. Scale bar is 200 nm. (**K**–**O**) STM images of nanoribbons after exposure times of 6, 19, 46, 72, and 98 min (applied bias  =  2 V, tunneling current  =  0.1  nA). Scale bar is 100 nm. Color is scaled to topographic height, with dark red being lowest and light yellow being highest. Reproduced from Ref. [106].

Regardless of substrate, successful ribbon formation relies on pronounced kinetic asymmetry in which the axial growth velocity greatly exceeds the lateral velocity, as seen in Figure 4A–E [111]. On Ge (001), selective C-Ge bonding along ⟨110⟩ facets promotes armchair-edge propagation while suppressing side attachment, as evident from Figure 4F–O [106,107]. In hydrogen-moderated liquid-Cu systems, a tuned H_2_ flux preferentially etches lateral sp^2^ edges while preserving elongation [110]. This anisotropy stabilizes narrow, self-limiting ribbons and delays coalescence into two-dimensional films. The electronic consequence parallels the geometric one: as Son, Cohen, and Louie showed, armchair edges produce width-dependent semiconducting bandgaps, whereas zigzag edges host spin-polarized states [112].

Uniform nucleation is equally critical for dimensional control. Without regulation, ribbons nucleate stochastically, which leads to broad width distributions. Seed-assisted growth plays a central role in controlling graphene nanoribbon nucleation on Ge(001). As demonstrated in [106], PAH molecules such as PTCDA and pentacene act strictly as nucleation seeds, defining where growth begins and ensuring synchronous ribbon initiation, but they do not lower the activation energy for C–C bond formation. Their function is limited to setting spatially uniform nucleation sites, which reduces stochastic variability in ribbon width and spacing. Building on this principle, Shekhirev et al. extended seed-based growth to lower temperatures, showing that thermal decomposition of PTCDA between 500 and 650 °C produces oxygen-functionalized nanoribbons directly on insulating substrates, enabling immediate device operation as gas sensors without transfer steps [113].

### Substrate–Template Synergy

Recent advances in graphene nanoribbon (GNR) synthesis demonstrate that geometric confinement and surface-directed growth can complement chemically driven anisotropy. One example is the use of pre-etched monolayer-deep h-BN trenches, typically a few to several nanometers wide, which constrain CVD growth along crystallographically defined zigzag directions. Because the graphene forms inside the trenches, these structures provide built-in dielectric encapsulation and eliminate the need for post-growth transfer steps [114].

On germanium substrates, anisotropic growth on Ge(001) surfaces produces elongated armchair-oriented ribbons whose aspect ratios arise from the strong dependence of growth velocity on the relative alignment between the graphene armchair direction and Ge(110) surface lattice vectors [111]. Under seeded growth conditions, polycyclic aromatic hydrocarbons such as PTCDA or pentacene act as nucleation seeds, enabling more uniform and synchronous ribbon initiation while suppressing spontaneous methane-driven nucleation [106].

Distinct from Ge-based growth, hydrogen-regulated etching on liquid copper creates comb-like channels that guide graphene growth without lithography. Hydrogen selectively etches surface features to generate parallel channels, producing highly aligned, micrometer-scale graphene ribbons, including sub-10 nm structures formed through balanced growth and etching dynamics [110]. These approaches demonstrate that physical confinement, surface reconstruction, and gas-mediated etching can each be tuned to promote directional growth and suppress film coalescence.

The electronic behavior of the resulting ribbons follows well-established edge-structure principles: armchair GNRs exhibit width-dependent semiconducting bandgaps, whereas zigzag edges support spin-polarized states, as described by the theoretical framework provided in the uploaded reference on GNR electronic structure [112].

Beyond high-temperature growth on Ge or Cu, lower-temperature routes have also been demonstrated. Thermal decomposition of PTCDA between 500 and 650 °C produces oxygen-terminated armchair GNRs directly on insulating substrates, enabling immediate device fabrication. These films exhibit ambipolar response and strong sensitivity to a range of volatile organic compounds, allowing their use as GNR-based gas sensors without transfer or high-vacuum processing [113].

Finally, post-growth thermal annealing of GNRs at temperatures up to 2800 °C induces significant structural reconstruction, including the healing of point defects and transformation of open edges into single-, double-, and multi-loop edge closures, as confirmed by transmission electron microscopy and Raman spectroscopy. These reconstructions reduce disorder and improve graphitic ordering, illustrating how thermal processing can refine the structural quality of nanoribbons after growth [104].

Recent CVD studies highlight that although anisotropic growth on Ge(001) reliably produces sub-10 nm armchair-edge graphene nanoribbons with high alignment and aspect ratios above 10 [107], controlling width uniformity remains challenging. Experiments show that growth rate strongly affects lateral expansion, with slower growth (achieved through higher H_2_:CH_4_ ratios) producing more elongated shapes, whereas faster growth leads to broader, less anisotropic structures [107].

Complementary kinetic Monte Carlo modeling indicates that anisotropic ribbon formation requires direction-dependent stabilization of graphene precursor species and anisotropic activation energies for row nucleation and row growth [108]. These models also reveal that the diffusion length of precursor species governs the transition between high-aspect-ratio growth and lateral widening [108].

Beyond Ge(001), hydrogen-regulated etching on liquid copper produces comb-like channels that guide growth and enable large-area aligned GNR arrays, where hydrogen flow rate strongly controls channel width and uniformity [110]. Similarly, seed-defined nucleation using polycyclic aromatic hydrocarbons enables synchronized ribbon initiation and suppresses uncontrolled nucleation on Ge(001), improving reproducibility [106]. Taken together, these studies demonstrate that while high-aspect-ratio ribbons are achievable, precise width control remains highly sensitive to growth rate, precursor diffusion, hydrogen etching dynamics, and nucleation density. Continued development of predictive models that couple nanoscale mechanisms to macroscopic growth parameters is needed to transition from empirical optimization to fully controlled CVD synthesis.

Having examined the anisotropic, substrate-guided growth of GNRs, the discussion now advances toward CNWs, vertically oriented, few-layer graphene architectures that embody the transition from lateral confinement to field-directed vertical assembly. This dimensional progression from ribbon to wall highlights how CVD growth can be reoriented from surface-limited kinetics to plasma-assisted, self-organized frameworks.

## 7. Carbon Nanowalls (CNWs)

CNWs, vertically aligned, few-layer graphene networks, represent one of the most distinctive nanocarbon architectures attainable through CVD. These combine the high electrical conductivity of graphene with the exceptional surface-to-volume ratio of porous graphitic networks, positioning them as multifunctional materials for catalysis, energy storage, sensing, and protective coatings.

**Figure 5:**
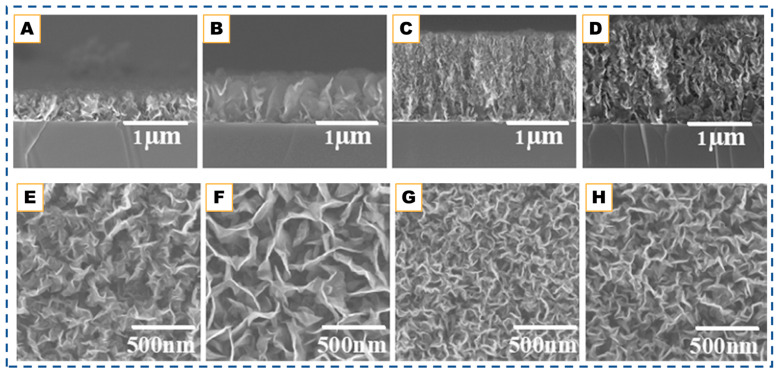
SEM sectional (**A**–**D**) and top-view (**E**–**H**) images of VG which are deposited at 600 °C but with different plasma powers, i.e., 100 (**A**,**E**), 200 (**B**,**F**), 300 (**C**,**G**), and 400 W (**D**,**H**), respectively. Reproduced from Ref. [116].

The origin of CNW research can be traced back to as early as 2002 [34]. The study demonstrated that carbon radicals in plasma environments self-organize into vertically aligned carbon walls on sapphire due to charge buildup on the insulating substrate, which generates strong transverse electric fields that direct CNW growth instead of CNTs. Subsequent study expanded the understanding of CNW synthesis by showing that well-defined surface-bound structures could be formed on plain silicon without metal catalysts, while freestanding CNWs fabricated on metallic supports confirmed their mechanical robustness and scalability, proving that vertical graphene frameworks could be detached and handled without collapse [115]. Together, these studies established CNWs as a field-directed, plasma-grown carbon system, where morphology is governed by a triad of carbon addition, hydrogen etching, and electric-field alignment. Building on this foundation, subsequent research explored remote PECVD for the controlled fabrication of vertical graphene (VG), where the growth direction and structural characteristics are tuned by adjusting plasma density, growth temperature, and ratio between carbon source and carrier gas, as shown in Figure 5 [116].

Further mechanistic clarity emerged from high-resolution TEM studies. TEM images revealed that nucleation initiates at mismatched or curved graphitic layers within a thin amorphous-carbon buffer layer on the substrate, or alternatively from carbon onions whose outer shells provide defective graphitic curvature that seeds upright sheets. Once nucleated, the walls extend vertically through the diffusion of carbon adatoms along the graphene surfaces toward active step edges, where attachment governs the growth rate. The characteristic curved, terraced steps observed in TEM directly map onto mass-flow simulations, confirming that carbon adatom concentration gradients drive upward propagation. Growth ultimately terminates when adjacent graphene layers merge to form seamless, closed edges, explaining the tapered morphology and finite height of CNWs and VG structures [117].

Most recently, efforts to expand CNW synthesis toward temperature-sensitive and flexible substrates have led to the development of radical-injection PECVD (RI-PECVD) routes capable of achieving catalyst-free vertical growth at temperatures as low as 225 °C, a significant reduction from the conventional 600–800 °C range required in earlier PECVD systems. Systematic SEM, Raman, and TEM analyses revealed that CNW formation at this ultra-low temperature proceeds through the same four-stage sequence established for high-temperature growth—nano-island formation, edge-site nucleation, vertical sheet emergence, and wall thickening—yet with reduced precursor fluxes and radical diffusivities that slow the initiation stages. Importantly, despite the lower processing temperature, the resulting CNWs exhibit multilayered graphene walls, high verticality, and exceptionally high wall densities, which enhance hydrophobicity and surface roughness. This work demonstrates that the fundamental field-assisted, radical-driven mechanism is preserved even at low thermal budgets, enabling CNW synthesis on broader classes of substrates and opening pathways for scalable, low-temperature device integration [118].

Parallel advances have also targeted architectural control of CNWs through multistep plasma growth. A two-stage RI-PECVD and CCP-CVD process was shown to produce hierarchical multi-branched CNWs with wall densities and nanoscale spacing unattainable in single plasma modes. In this scheme, RI-PECVD first creates vertically aligned graphene walls with abundant edge sites, while the subsequent low-power CCP step, with reduced hydrogen etching and modified CH and C_2_ radicles chemistry, induces lateral nucleation and branching. Time-resolved SEM and TEM reveal that carbon nuclei form on wall tops and evolve into secondary nanosheets, yielding a high-surface-area superhydrophobic CNW network. Although the core vertical growth mechanism still relies on the balance between carbon addition and hydrogen etching, this dual-regime approach offers enhanced morphological tunability for sensing, catalytic, and surface functional applications [119]. More recently, a comprehensive plasma physics perspective [120] clarified why different PECVD methods yield such diverse CNW morphologies. Systematic analysis of ion and radical interactions shows that CNW growth is sustained only within a narrow window of ion energy and ion flux, where bombardment is sufficient to activate edge sites yet not strong enough to amorphized the carbon network. This framework explains why remote, inductively coupled, and radical injection plasma systems outperform conventional CCP discharges: they decouple ion energy from radical density, allowing independent control over defect content, sheet verticality, nucleation rate, and wall density. The review further demonstrates that electric field strength in the plasma sheath remains the primary factor enforcing vertical alignment, as alterations to the substrate field environment can switch growth from vertical to planar. These insights provide a unifying mechanistic foundation that links historical observations with modern PECVD strategies and clarifies why plasma configuration, rather than temperature alone, governs CNW architecture [120].

A complementary direction has focused on using substrate geometry to guide CNW arrangement. Nanoporous anodic alumina templates have shown that pore diameter and membrane thickness regulate nucleation density, wall spacing, and growth direction. Larger pores promote well-separated vertical walls, whereas smaller or curved pore edges trigger mixed horizontal and vertical nucleation as flakes merge across confined regions. Substrate thickness further influences wall height by modifying local thermal and electrical conductivity. Although this approach does not alter the plasma-driven mechanism itself, it demonstrates that surface topography can imprint spatial order onto CNW assemblies, providing an additional route to control density and architecture for patterned or conformal vertical graphene networks [121].

From a heteroatom-doping standpoint, post-treatment nitrogen incorporation has emerged as an effective route to enhance the electrochemical activity of carbon nanowalls [122]. Direct current plasma enables the introduction of pyridinic, pyrrolic, and graphitic nitrogen species into the graphene edges and vacancy sites, where their lone-pair and π-electron interactions create highly accessible redox-active centers. Although the total nitrogen content remains modest, the resulting defect-rich surface dramatically increases specific capacitance, rising from about 105 F g^−1^ in pristine CNWs to nearly 600 F g^−1^ after doping, due to improved charge transfer, wettability, and pseudocapacitive contributions. DFT analyses further show that nitrogen binds preferentially at vacancy-rich regions, confirming that doping stability and electrochemical enhancement are governed by defect chemistry rather than bulk nitrogen concentration. Together, these findings highlight heteroatom doping as a practical and powerful means to transform CNWs from passive, vertically aligned carbon architectures into highly active electrode materials suitable for micro-supercapacitors and other electrochemical devices [122].

In parallel with electrochemical enhancements achieved through heteroatom doping, other studies have examined the intrinsic transport properties of CNWs to assess their suitability for microelectronic and sensing platforms. CNWs synthesized on platinum thin films and interdigitated electrodes exhibit well-defined interface-dependent behavior, transitioning from Schottky-like rectification on Pt layers to nearly linear, graphene-like conduction on patterned electrodes. Hall measurements reveal p-type conductivity with a high carrier concentration, while comparative analyses of different Ar flow conditions show that plasma stability influences wall density and sheet thickness. Post-growth Ar/N_2_ exposure further modifies conductivity through controlled thinning of the graphitic walls. These interface- and morphology-dependent electrical characteristics complement electrochemical findings by highlighting how substrate selection, plasma stability, and post-treatment shape the transport pathways essential for CNW-based devices [123].

Mechanical characterization remains less explored but equally vital. Nanoindentation analysis reported a Young’s modulus value of ~28 GPa and compressive strength near 50 MPa, revealing elastoplastic deformation and confirming that CNW films, while robust, remain susceptible to delamination on smooth substrates. These results reinforce the need for graded interfaces or textured templates to enhance adhesion and durability [124].

Across early and contemporary studies, a consistent narrative takes shape: [34,115,119] plasma assistance provides both the kinetic freedom and the field-induced directionality required for catalyst-free, self-organized vertical graphene growth. In practice, PECVD has become the most widely adopted route because several advantages converge. First, its broad temperature window enables vertically aligned CNWs to be synthesized at temperatures as low as ~225 °C, far below the >700 °C typically required in thermal CVD, thereby extending compatibility to polymeric and CMOS-relevant substrates [118]. Second, PECVD platforms decouple radical generation from ion-driven etching, allowing systematic adjustment of crystallinity, defect density, and wall spacing through control of plasma density and ion energy [120]. Third, large-area uniformity has been demonstrated, with freestanding CNW films retaining vertical alignment over centimeter-scale regions [115]. Building on this foundation, dual-source configurations such as RI-PECVD further refine growth control by separating radical production from ion acceleration, enabling low-damage growth while maintaining vertical order [120]. Collectively, these developments show that PECVD effectively integrates controlled energy delivery, field-directed sheet orientation, and a tunable balance between deposition and etching, establishing it as the central methodology for CNW synthesis.

Despite these advances, PECVD-grown CNWs face several intrinsic constraints. The most prominent is the temperature-defect trade-off: low-temperature growth increases compatibility with flexible substrates but yields higher defect densities, as reflected by elevated Raman D bands and reduced graphitic ordering [118]. These defect-rich structures can enhance catalytic and capacitive behavior but degrade conductivity and mechanical strength. Excessive plasma power or exposure time can also promote sidewall deposition and branching, disrupting verticality and uniformity [119]. At a more fundamental level, the process itself remains only partially mapped: while parameters such as pressure and high-frequency power can modulate wall density, other key microstructural descriptors such as defect index, crystallinity, and edge character show minimal or unpredictable response to these inputs. The CH_4_/H_2_ study highlights this limitation, noting that CNW growth is governed by complex interactions among CH_x_ and H radicals, that diagnostic tools capture only part of this chemistry, and that no unified growth mechanism or quantitative process structure framework yet exists. This lack of comprehensive correlations continues to restrict predictive control and reproducibility in PECVD systems [125].

Recent analyses [120] have emphasized that CNW growth mechanisms remain incompletely understood, with ion–radical interactions and flux balances still poorly quantified. These studies highlight the need for real-time plasma diagnostics and integrated kinetic modeling to achieve predictive control in PECVD systems.

After examining vertically aligned CNWs, the dimensional evolution of CVD-grown carbon continues toward fully interconnected three-dimensional architectures. GFs represent this next stage, where lateral continuity and vertical alignment merge into continuous porous networks that extend the principles of plasma- and template-assisted growth into macroscopic, freestanding forms.

## 8. Graphene Foams (GFs)

The emergence of 3D GFs represents one of the most transformative developments in the chemistry of carbon materials. Within just over a decade, the field has progressed from serendipitous template replication to deliberate architectural design, driven primarily by advances in CVD. These interconnected graphene frameworks have evolved from scientific curiosities into functional materials that now serve as conductive scaffolds for energy devices, mechanical reinforcements in composites, and programmable architectures in their own right.

**Figure 6:**
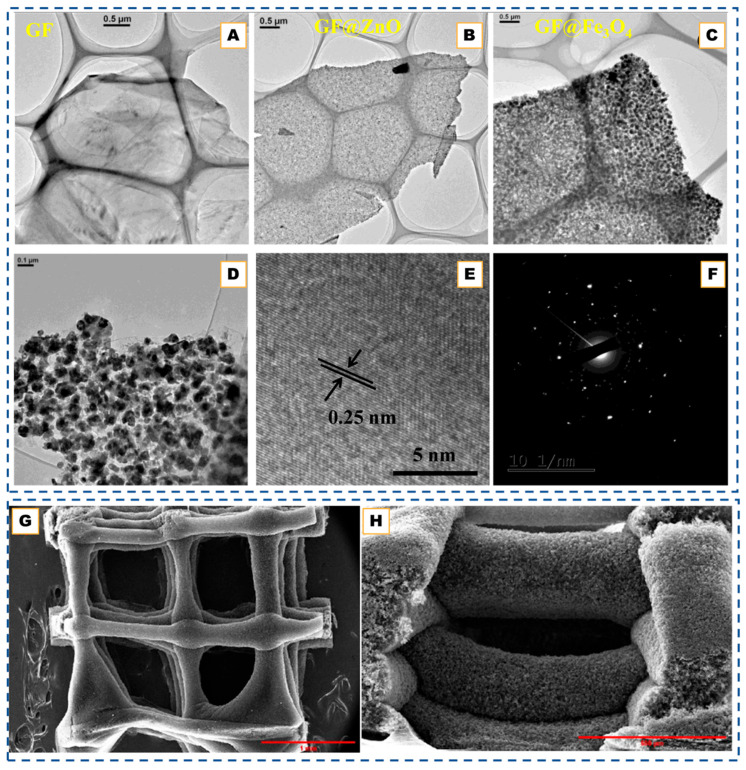
TEM Images of GFs. (**A**) GF. (**B**) GF@ZnO. (**C**) GF@Fe_3_O_4_. (**D**) HRTEM of an individual Fe_3_O_4_ nanoparticle. (**E**) SAED patterns of GF@ Fe_3_O_4_. (**F**) Reproduced from Ref. [127]. Copyright 2013 American Chemical Society. (**G**) Top view of the sample used for SEM analysis with a 1 mm scale bar; (**H**) side view of the sample used for SEM analysis with a 500 μm scale bar. Reproduced from Ref. [131].

Graphene’s exceptional electrical, thermal, and mechanical properties were long constrained by its intrinsic planarity. The translation of atomically thin layers into self-supporting 3D architectures was, therefore, both a conceptual and technological breakthrough. In 2011, Chen et al. demonstrated that methane decomposition on nickel foam at atmospheric pressure produced a freestanding graphene network after template removal [126]. This foam, with an ultralight density of about 5 mg cm^−3^, a surface area near 850 m^2^ g^−1^, and scalable dimensions up to 170 × 220 mm^2^, established template-assisted CVD as a reproducible route to continuous, macroscopic graphene. Soon after, Luo et al. incorporated a bicontinuous mesoporous Fe_3_O_4_ nanostructure into graphene foams (Figure 6A–F), producing flexible, high-rate lithium-ion battery anodes with an outstanding performance, including stable cycling over 500 cycles and capacities up to 190 mAh g^−1^ at 60C [127]. These early studies confirmed that CVD-grown graphene maintains electronic continuity across complex 3D topologies, a performance that reduced-oxide sponges and sol–gel aerogels could not match.

By the mid-2010s, the field had settled into a coherent taxonomy in which three-dimensional graphene foams, sponges, and aerogels were understood as distinct families of porous carbon architectures. It was widely recognized that only CVD-grown foams exhibit seamless, continuous graphene networks, whereas structures assembled from graphene oxide rely on percolated flakes with intersheet junctions. Nevertheless, several fundamental weaknesses remained: uncontrolled multilayer growth on nickel scaffolds, spatial non-uniformity inherited from the metal template, and frequent structural collapse during template etching or drying, problems especially severe in monolayer systems. These shortcomings set the stage for the next phase of progress, centered on tighter process control and improved morphological stability [128,129].

One promising response to these challenges emerged with the development of engineered microcellular nickel templates, which offered far greater thermal and mechanical stability during CVD [130]. By introducing an electropolishing-assisted electroless plating route, this work enabled the fabrication of millimeter-thick, uniformly structured Ni scaffolds with 1–100 μm pores that could withstand high-temperature growth without the shrinkage or collapse that plagued earlier foams. These templates allowed the resulting graphene frameworks to retain their architecture after metal removal, substantially improving morphological fidelity. Yet, despite this progress, several intrinsic limitations of Ni-templated CVD persisted. The graphene walls remained uncontrolled few-layer stacks, and nanoscale uniformity in thickness could not be fully imposed, leaving the broader issues of multilayer growth and atomic-scale consistency unresolved. Thus, while template stability and macroscopic uniformity advanced significantly, the fundamental challenges associated with layer control in CVD-grown graphene foams continued to motivate further innovation.

### 8.1. Reforming the Format: Film-Type and Cast-Catalyst Foams

The next major shift came with the transition from bulk metal foams to metallurgical films capable of supporting graphene growth. Building on the limitations of commercial Ni foam, Huang and co-workers reformulated the catalyst into thin porous Cu/Ni films whose pore sizes, alloy composition, and thickness could be precisely tuned before CVD. These films enabled the growth of mechanically robust, few-layer graphene foams with markedly smaller pores, extending down to the sub-micrometer scale, while retaining the continuous electronic pathways characteristic of CVD graphene. Unlike earlier Ni-foam replicas, these film-type architectures survived template removal without polymer reinforcement and delivered properties inaccessible to bulk templates, including high electrical conductivity, strong hydrophobicity, rapid organic-solvent uptake, and exceptionally high EMI shielding performance. In effect, the shift to Cu/Ni films reframed 3D graphene not as a product of serendipitous templating, but as an engineered coating conformally grown on a customizable metallurgical scaffold [10]. In parallel, a complementary format emerged through the development of castable catalyst films, which departed entirely from the foam paradigm. Instead of relying on pre-made metal foams or alloy films, this method dispersed Ni powder into a polymer slurry that could be doctor-bladed or mold-cast into arbitrarily large sheets. Upon CVD treatment and subsequent removal of the Ni, the composite yielded flexible, freestanding three-dimensional graphene sheets whose porosity, thickness, density, and conductivity could be tuned simply by adjusting slurry composition and compression conditions. This approach overcame long-standing barriers to scalability by eliminating the size, cost, and tooling constraints associated with sintered or commercial Ni foams, enabling meter-scale production while maintaining structural integrity through high-temperature growth and aggressive etching. Yet, even this innovation did not resolve the intrinsic multilayer nature of Ni-catalyzed graphene: the resulting networks remained composed of several-layer shells with limited control over atomic thickness. Nevertheless, cast-catalyst processing represented an essential broadening of the 3D graphene design space, transforming foams into manufacturable films suited for continuous fabrication and large-area devices [132].

### 8.2. Designing Geometry: The Rise of Additive Manufacturing

In the following years, additive manufacturing introduced intentional design to what had once been a templating convenience. Du et al. employed digital light processing to print gyroidal silica ceramic templates, which were subsequently conformally coated by CVD and etched to yield graphitic gyroid lattices capable of supporting approximately 16,000 times their own weight [133]. This fusion of AM and CVD enabled mechanically programmed 3D graphite/graphene foam, where unit-cell geometry controls stiffness and strongly influences electrochemical transport behavior. A complementary approach by Kondapalli et al. used direct-ink-written Ni-PLGA composites as self-catalyzing templates for atmospheric-pressure CVD, forming binder-free graphene lattices with conductivities around 17 S cm^−1^ as seen in Figure 6G,H [133]. Their method eliminated commercial metal foams entirely, demonstrating that the catalyst and structure can be co-printed as a single functional entity. Ramírez et al. expanded substrate versatility by demonstrating catalyst-free CVD of nanocrystalline graphene directly on 3D-printed alumina lattices [134]. Eliminating metal catalysts, etching, and transfer steps allowed graphene to be integrated with robust ceramic frameworks, enabling high-temperature and load-bearing architectures beyond the thermal and mechanical limits of conventional graphene foams.

### 8.3. From Empiricism to Design: Understanding and Integration

By 2022, graphene-foam research was moving toward design-oriented synthesis. Reviews of CVD-derived 3D graphene highlighted how templating routes, carbon sources, and catalyst choices govern pore connectivity, wall thickness, and overall architecture, positioning template-assisted CVD as a tunable method for producing high-porosity multilayer graphene networks and identifying emerging directions such as plasma-assisted growth and alternative precursors [135]. Conceptual work on continuous porous architectures emphasized that minimal-surface-like curvature, pore size, and topology can be used to relate 3D structure to electronic, catalytic, and mechanical behavior [135].

Multiscale simulations incorporating atomistic and continuum models then clarified how deformation mechanisms in CVD-templated graphene foams vary with relative density and ligament morphology, revealing a shift from brittle to ductile behavior as ligaments become thinner and more flexible [136]. Collectively, these developments signal a transition from empirically inherited templates toward a more mechanistic and design-aware approach in 3D graphene foams, where architecture and properties are intentionally linked through controlled synthesis and modeling [135,136].

## 9. Emerging Patterns and Unresolved Questions

Chronologically, the development of 3D GFs unfolds in three interconnected phases. The discovery phase (2011–2015) marked the establishment of Ni-foam-based CVD as a practical route for producing freestanding 3D graphene networks. Early studies demonstrated that graphene could grow conformally over commercial Ni foams to form continuous, bicontinuous porous architectures rather than isolated flakes or stacked films. These works established the experimental feasibility and foundational terminology of CVD-grown 3D graphene foams [126,127].

The refinement phase (2016–2020) focused on improving microstructural control, scalability, and morphology. Advances included the use of electrodeposited or electroless Ni templates, improved control over pore size and ligament thickness, and enhanced graphitic quality achieved through optimized APCVD conditions. These efforts produced foams with more uniform pores, thinner graphitic walls, and greatly improved structural stability and diffusion characteristics, effectively transforming 3D graphene from a proof-of-concept into a tunable platform for electrochemical and functional applications [10,129,132].

The design phase (2021–2025) has elevated synthesis into an exercise in architectural engineering. Additive manufacturing has enabled geometries far beyond the constraints of metal foams, while catalyst-free CVD on 3D-printed ceramics has introduced new high-temperature, structurally robust architectures. In parallel, multiscale modeling has clarified deformation mechanisms in CVD-templated graphene foams, linking relative density, ligament morphology, and mechanical response. Together, these developments signify a shift from empirical template inheritance toward computationally and architecturally guided synthesis [131,133,134]. Despite this coherent trajectory, several critical challenges persist. Quantitative relationships between CVD parameters, such as precursor ratios, temperature gradients, and cooling dynamics, and the resulting graphene quality within complex 3D geometries remain inconsistent, limiting reproducibility across laboratories. The interfacial durability of graphene integrated with metallic or ceramic scaffolds under realistic mechanical, thermal, and electrochemical conditions is still poorly understood. Moreover, while multiscale models capture macroscopic deformation, these seldom incorporate the chemical kinetics of carbon deposition, leaving defect evolution and layer continuity unresolved.

The absence of continuous or high-throughput manufacturing routes, including roll-to-roll and rapid-heating CVD systems, further constrains industrial scalability. Overcoming these limitations will determine whether 3D GFs remain confined to academic exploration or emerge as foundational materials for next-generation energy, structural, and electronic technologies.

## 10. Comparative Insights in Morphological Transitions in CVD-Derived Carbon Nanostructures

**Figure 7:**
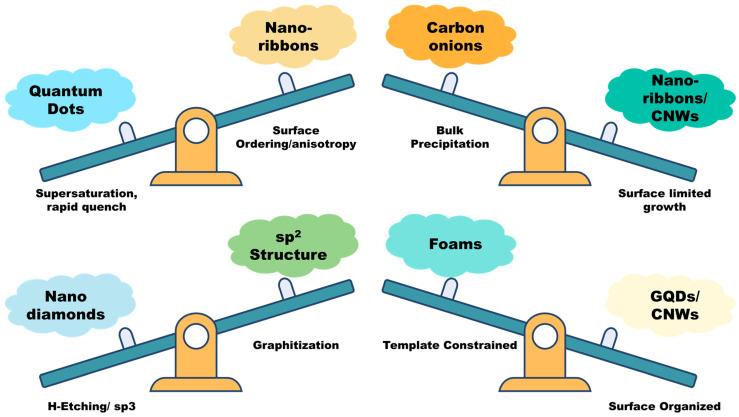
Conceptual “control-lever” schematic illustrating the competing kinetic and thermodynamic parameters that steer morphology evolution during CVD growth of carbon nanostructures. Each lever represents an opposing regime whose balance determines the final structure: (**top left**) supersaturation and rapid quenching favor quantum dots, while surface ordering and anisotropy yield nanoribbons; (**top right**) bulk precipitation produces carbon onions, whereas surface-limited growth forms nanoribbons and CNWs; (**bottom left**) hydrogen etching stabilizes sp^3^ nanodiamonds, opposing graphitization toward sp^2^ architectures; (**bottom right**) template-constrained growth leads to structured foams, while surface self-organization drives the formation of GQDs and nanowalls.

Having discussed the evolution of various carbon morphologies under different CVD conditions, it is useful to consolidate these observations (Figure 7). The morphological diversity observed in CVD-grown carbon nanostructures arises from a balance between competing thermodynamic and kinetic parameters, namely carbon supersaturation, surface diffusion, etching, and geometric confinement. As illustrated in Figure 7, these parameters act as a “morphological balance” that determines whether carbon condenses into 0, 1, 2, or 3D architectures. When nucleation dominates over diffusion, high supersaturation and rapid cap closure favor zero-dimensional forms such as QDs or CNOs. Increasing surface diffusion or directional activation promotes anisotropic growth, shifting the balance toward nanoribbons, vertical graphene, or CNWs. At the opposite extreme, template-guided conformal deposition and balanced etch-growth kinetics yield three-dimensional graphitic foams. This continuum reflects a dynamic interplay between supersaturation, diffusion, and confinement, where the “tilt” of the balance governs both morphology and bonding character, from sp^3^-rich nanodiamonds or CQDs to sp^2^-dominated ribbons, walls, and foams. Intermediate regimes, such as carbon onions and GQDs, represent transitional structures combining nucleation-driven and surface-directed growth. Thus, tuning these CVD control levers, such as chemical potential, diffusion, etching, and confinement, provides a unified framework for directing carbon nanostructure synthesis and for navigating morphological evolution across the CVD landscape, from dots to onions to ribbons to foams.

## 11. Comparative Spectroscopic and Structural Characterization of CVD-Derived Carbon Nanostructures

The evolution of carbon dimensionality, from 0D quantum dots to 3D graphitic foams, is mirrored by systematic transitions in Raman vibrational fingerprints, XPS chemical states, and XRD diffraction coherence. Integrating these three probes provides a reliable diagnostic framework for identifying and distinguishing among diverse CVD-grown nanocarbons.

### 11.1. CQDs, CNDs, and GQDs

**Figure 8:**
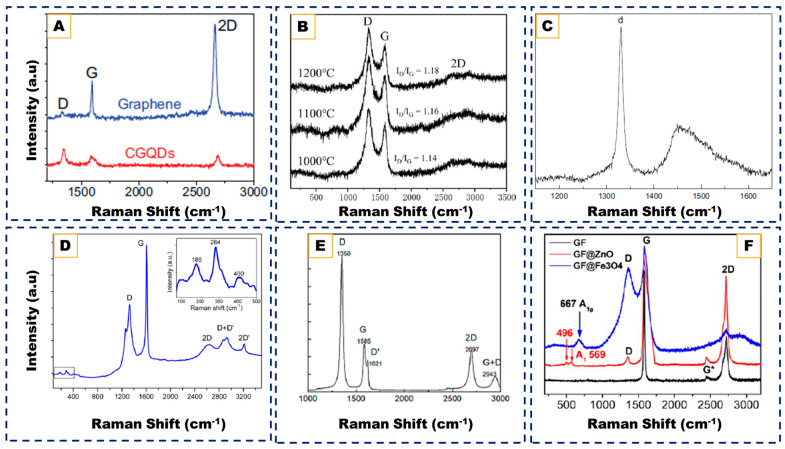
(**A**) Raman spectra of GQDs, reproduced with permission from Ref. [41]. Copyright 2013 WILEY-VCH Verlag GmbH & Co. KGaA, Weinheim. (**B**) Raman spectra of SiC@G core–shell nanoparticles prepared at different temperatures. Reproduced with permission from Ref. [69]. Copyright 2020 The American Ceramic Society. (**C**) Typical Raman spectrum obtained with a 473 nm laser from which the diamond Raman peak labeled by the letter “d” is visible together with a broader band attributed to transpolyacetylene. Reproduced with permission from Ref. [88]. Copyright 2019 WILEY-VCH Verlag GmbH & Co. KGaA, Weinheim. (**D**) Raman spectra of GNRs transferred on fused silica, measured at 532 nm; the inset is the magnified low-frequency mode (black oblong, bottom left). Reprinted with permission from Ref. [139]. Copyright 2016 American Chemical Society. (**E**) Raman spectrum of vertical graphene (CNWs). Reproduced from Ref. [116]. (**F**) Raman spectra of the GF, GF@ZnO, and GF@Fe_3_O_4_ nanostructures. Reproduced with permission from Ref. [127]. Copyright 2013 American Chemical Society.

Raman spectra of carbon dots are dominated by disorder-activated D bands (~1340–1360 cm^−1^) and graphitic G bands (~1580–1600 cm^−1^), accompanied by a weak or diffused 2D feature near 2700 cm^−1^, as seen in the Raman spectrum showing graphene and CQDs in Figure 8A for the high ID/IG ratio (≈1.2–2.0). The strong photoluminescent background originates from the nanometric domain size and abundant surface functionalities [137]. Upon annealing, ID/IG decreases and the 2D band sharpens, reflecting domain coarsening and defect healing. The respective XPS typically reveals a dominant sp^2^ C=C component (≈284.5 eV) plus intense C-O/C=O peaks (286–288 eV), signifying oxygenated edges and surface groups. Graphitization or reduction treatments suppress oxygen peaks and enhance the π–π* shake-up tail, confirming restoration of conjugation [138].

Their XRD patterns show a broad, low-intensity (002) reflection between 20 and 26° 2θ, indicative of short-range order and large interlayer spacing (0.345–0.360 nm). Progressive annealing sharpens this feature and shifts it to higher angles, aligning with the rise in sp^2^ fraction detected by XPS. Together, these data mark the transition from amorphous to nanocrystalline graphene domains [140].

### 11.2. CNOs: Solid, Hollow, and Core–Shell

CNOs exhibit Raman features of curved turbostratic graphite: balanced D (~1350 cm^−1^) and G (~1580 cm^−1^) bands and a broad asymmetric 2D band reflecting rotational disorder, as shown in Figure 8B. Solid onions show a slightly up-shifted, narrower G band due to compressive strain, while hollow forms display a down-shifted, broadened G caused by shell relaxation. core–shell CNOs (metal@C) may show additional weak modes or baseline changes from carbide phases [141]. Thermal annealing consistently lowers ID/IG and sharpens 2D, indicating stress release and ordering.

XPS spectra are sp^2^-dominated, with minimal oxygen components. Hollow shells, having a higher surface area, often retain more C–O/C=O groups, whereas filled onions display cleaner graphitic envelopes. For metal@C systems, survey spectra confirm metal or carbide signals that diminish after etching [142].

XRD presents a broad (002) reflection near 25–26° 2θ (d ≈ 0.338–0.342 nm). Hollow or defective shells may shift the peak slightly to lower angles (expanded spacing), while high-temperature treatment restores it toward graphite values. The (100)/(101) in-plane bands emerge subtly with improved shell order, correlating with TEM lattice-fringe regularity [143].

### 11.3. NDs

Raman spectra of nanodiamonds are distinguished by the sp^3^ diamond line at 1332 cm^−1^, often superimposed on a D/G background from surface graphitic layers, as shown in Figure 8C. The I_1332_/(D + G) ratio and FWHM of the 1332 cm^−1^ line gauge crystalline purity and strain.

In XPS, C 1s deconvolution reveals overlapping sp^3^ (~285 eV) and sp^2^ (~284.5 eV) contributions. Purified or oxidized NDs show higher sp^3^ fractions and diminished oxygenated peaks; hydrogen termination further reduces O content. These chemical trends parallel the sharpening of the diamond Raman peak [144].

The respective XRD patterns typically show broad diamond reflections, (111) at ~44° and (220) and (311) at higher 2θ, consistent with nanocrystalline cubic carbon. A weak graphitic (002) may appear if surface shells persist. Oxidative cleaning suppresses this sp^2^ peak, confirming successful purification [145].

### 11.4. GNRs

GNRs exhibit edge-activated D bands (~1345–1355 cm^−1^) even in high-quality samples, a hallmark of finite-width confinement, as shown in Figure 8D. The ID/IG ratio scales inversely with width; narrower ribbons yield higher D intensity. The 2D band weakens and broadens, while the D′ (~1620 cm^−1^) assists in separating edge from basal defects. Polarized Raman mapping reveals orientation-dependent D/G ratios, indicating edge alignment [146].

XPS captures sp^2^-dominated C 1s with small sp^3^ and oxygenated components concentrated at edges. In doped ribbons, N 1s spectra resolve pyridinic (~398.5 eV), pyrrolic (~400.1 eV), and graphitic (~401.0 eV) sites; their ratios describe doping topology [108].

In most studies, isolated or well-dispersed GNRs exhibit very weak or diffuse XRD features because of limited stacking coherence. When the ribbons are assembled into stacked or transferred films, a broadened graphitic (002) reflection typically appears near 2θ ≈ 26°, signifying partial restacking of basal planes. Upon thermal annealing or chemical reduction, this (002) feature often becomes sharper and more intense, consistent with a modest decrease in the Raman ID/IG ratio and an increase in the sp^2^ carbon fraction observed in XPS, reflecting progressive structural ordering [145].

### 11.5. CNWs

Vertically aligned CNWs, few-layer graphene sheets standing on substrates, display Raman spectra with strong D and broad 2D bands, typical of high edge density and turbostratic stacking, as evident in Figure 8E. As growth temperature or hydrogen etching increases, ID/IG decreases and the G band narrows, denoting improved sp^2^ reorganization. Post-annealing (>900 °C) further sharpens 2D bands and relaxes G position [147].

XPS analyses of CNWs generally reveal a mixture of sp^2^ and sp^3^/defect-like carbon, together with oxygenated surface species originating from plasma-induced edge functionalization and residual gas adsorption during growth. Increasing the substrate temperature or applying post-annealing treatments typically reduces the oxygen content and enhances the relative sp^2^ fraction, reflecting improved structural ordering. In heteroatom-doped CNWs, additional N 1s, B 1s, or P 2p components are commonly detected, confirming dopant incorporation and providing insight into catalytic or electronic activity.

X-ray diffraction of CNWs usually exhibits a broad, low-intensity (002) reflection around 2θ ≈ 25°, characteristic of short stacking coherence and turbostratic ordering. With higher growth or annealing temperatures, this feature often becomes slightly sharper and shifts toward ~26°, indicating partial improvement in interlayer registry. Such structural evolution is consistent with the corresponding Raman signatures of reduced defect density and the increased sp^2^ content observed by XPS [148].

### 11.6. Graphitic/Graphene Foams

Three-dimensional GFs and aerogels consist of interconnected graphene walls forming continuous porous networks. Raman spectra reveal clear D and G bands with ID/IG ≈ 0.7–1.3 and a broad single-component 2D band, as shown in Figure 8F. Thermal graphitization decreases ID/IG, sharpens the 2D band, and slightly up-shifts G due to strain relaxation and reduced doping [149].

XPS shows predominantly sp^2^ carbon with modest oxygenated species at wall junctions; O content declines upon annealing. For doped foams, XPS directly quantifies heteroatom incorporation and bonding types.

XRD exhibits a broad (002) peak at ~25–26° 2θ that sharpens and increases in intensity as walls thicken and ordering improves. The (100) reflection may emerge weakly at ~43°, confirming in-plane registry. Concurrent decreases in BET surface area and oxygen fraction corroborate densification [10]. To make these dimensionality-dependent trends more explicit, Table 2 below provides a concise comparison of the characteristic Raman, XPS, and XRD signatures associated with 0D, 1D, 2D, and 3D carbon architectures, highlighting how disorder, stacking, and crystallinity evolve across scales.

Across the carbon dimensional spectrum, disorder, stacking, and crystallinity evolve in a systematic and experimentally observable way. In 0D systems such as carbon dots and carbon nano-onions, Raman spectra show strong D bands and broad G/2D features, reflecting short-range order and curved or amorphous domains [138,140]. XPS typically reveals mixed sp^2^/sp^3^ bonding with abundant oxygenated groups, while XRD exhibits highly broadened (002) reflections indicative of limited stacking coherence [140,141]. As dimensionality increases to 1D graphene nanoribbons and 2D carbon nanowalls, spectroscopic coherence strengthens: nanoribbons display edge-activated D modes and width-dependent Raman signatures [146], while nanowalls show crystalline 0.34 nm graphene layer spacing and characteristic D/G features arising from vertical sheet edges [147,148]. Correspondingly, XPS becomes increasingly sp^2^-dominated and XRD begins to show the emergence of graphitic (002) order. In 3D graphitic networks and mixed sp^2^–sp^3^ architectures, XRD reveals sharper (002) and occasional (100) reflections, and XPS indicates higher sp^2^ fractions associated with more extended conjugation [150]. Together, these correlated signatures from Raman, XPS, and XRD provide a coherent framework for tracking structural evolution from highly disordered nanoscale domains to more ordered graphitic architectures.

Real-time diagnostics such as in situ Raman spectroscopy, optical emission spectroscopy (OES), and spectroscopic ellipsometry are becoming increasingly important for controlling CVD growth of graphitic carbon materials, including NDs, CNOs, CNWs, and related architectures. In situ Raman enables direct tracking of defect evolution, sp^2^/sp^3^ transitions, and nanocrystallinity development during diamond and carbon film growth. These capabilities are consistent with its established sensitivity to grain-boundary phases, trans-polyacetylene components, and crystallite size in nanocrystalline and ultrananocrystalline diamond films [151]. OES provides complementary information on plasma species such as CH, C_2_, and H, offering insight into precursor dissociation pathways that govern the growth of diamond, CNOs, and turbostratic carbon forms. In addition, in situ spectroscopic ellipsometry, which has recently been validated for monitoring refractive index, void fraction, sp^2^/sp^3^ content, and film thickness during the earliest stages of polycrystalline diamond growth, enables continuous assessment of coalescence, ND phases, and growth kinetics under realistic plasma conditions [152]. Taken together, these real-time tools shift CVD of carbon nanostructures from static, post-growth assessment toward active, feedback-controlled processing, thereby improving reproducibility while enabling finer tuning of crystallinity, stacking order, and defect incorporation across 0D–3D carbon architectures.

## 12. CVD Scalability

Roll-to-roll CVD has already demonstrated laboratory-scale yet industry-relevant success in the continuous fabrication of graphene films, beginning with early breakthroughs reported as early as 2010 [153]. Despite the sophistication of CVD in tuning microstructure, there are, to date, no public reports by 2024 of CVD being used to achieve roll-to-roll or meter-scale production of quantum-dot layers, nor of CVD directly depositing continuous QD-based functional films over meter-length flexible substrates. In contrast, large-area integration of graphene quantum dots is currently realized via their solution dispersibility and use in printable inks, rather than through CVD-grown QD layers [154].

For NDS and CVD diamond films, the main limitation has traditionally been substrate seeding, as ultrasonication and spin-coating only work for small sample areas [155]. Since 2023, however, ultrasonic spray coating and inkjet printing have emerged as laboratory-demonstrated but roll-to-roll–compatible-in-principle strategies that enable uniform nanodiamond seeding over large-area substrates, thereby supporting high-quality diamond growth without yet demonstrating continuous meter-scale roll-to-roll operation [156].

A similar shift toward scalable production is evident for GNRs: while roll-to-roll CVD growth of atomically precise ribbons is still not achievable, a major 2023 breakthrough demonstrated a freezing–rolling–compression method capable of producing ~1 kg of high-quality nanoribbons in a single batch [157], representing a substantial scale-up beyond previous laboratory yields and opening conceptual, not yet implemented pathways toward roll-based processing.

Among all carbon nanomaterials, one of the most advanced demonstrations of roll-to-roll plasma-based growth beyond graphene films has been achieved for vertical graphene (carbon nanowalls). A plasma-based roll-to-roll CVD process reported in 2022 enables continuous deposition of vertically oriented graphene nanosheets on a moving metal foil while simultaneously converting CO_2_/CH_4_ into syngas—a combined materials-growth and gas-conversion process, though not claimed to be net-energy-positive [158]. Finally, graphene foams have transitioned from laboratory demonstrations to commercial reality. Despite this progress, significant hurdles remain for true industrial scalability: maintaining film uniformity across meter-scale substrates is nontrivial due to spatial plasma variations; precursor gases such as methane and high-purity hydrogen/CO_2_ mixtures add substantial operational cost; compatibility with flexible, thermally sensitive substrates is limited by the ∼300–700 °C growth temperatures; and integrating continuous web-handling with precise thermal control remains challenging. Collectively, these constraints highlight that while roll-to-roll approaches for vertical graphene are technologically promising, further advances in process economics, substrate engineering, and thermal management will be necessary for widespread deployment. To highlight how CVD compares with other established synthesis routes, Table 3 summarizes key differences in process conditions, scalability, and suitability for industrial integration.

## 13. Real-World Device Integration Challenges

Translating lab-grown CVD nanostructures into real-world devices poses significant challenges. In particular, compatibility with standard CMOS process flows and device architectures is a critical concern for bridging the gap between lab-scale demonstrations and practical applications. Many conventional CVD routes for carbon nanomaterials require high temperatures, often between 600 and 1000 °C, to achieve high crystalline quality. Such temperatures exceed the thermal budget of back-end-of-line (BEOL) CMOS processing, which typically must remain below ~400 °C [164]. For example, graphene grown on metal foils at ~1000 °C must be transferred onto the target substrate, a sequence incompatible with patterned wafers, where high temperatures can damage interconnects or induce metal diffusion. These thermal constraints are accompanied by additional integration. Carbon nanomaterials grown under such conditions must be interfaced with metal contacts and dielectrics in a device stack, and poor adhesion, high contact resistance, or weak bonding to gate dielectrics can undermine their intrinsic advantages. Within this broader context, several 3D graphitic architectures, notably vertically oriented graphene nanowalls [158] and CVD-grown graphene foams [130], have drawn attention because they demonstrate meaningful progress toward scalable synthesis. However, despite their scalability, these architectures still rely on high-temperature or plasma-based CVD and produce vertical or porous 3D morphologies that are poorly aligned with the planar, lithographically defined geometries required for wafer-level microelectronics. As a result, when translating such materials to substrates, thermal windows, patterning strategies, and process flows that remain compatible with established microfabrication practices continues to be an unresolved challenge.

## 14. Heteroatom Doping and Long-Term Stability

Heteroatom doping with elements such as nitrogen, boron, sulfur, and phosphorus is a widely used strategy to tailor the electronic, catalytic, and interfacial properties of carbon nanomaterials. However, despite its effectiveness in enhancing activity, the long-term stability of doped carbon frameworks remains a critical and still insufficiently understood issue, particularly in energy storage and electrocatalysis. Recent advances in nanoconfined and microenvironment-controlled catalysis highlight how dopant coordination, bonding, and local chemical surroundings strongly influence both performance and durability [165]. Doping introduces beneficial active sites, yet these same modifications can alter the chemical robustness and structural integrity of the carbon network over extended operation. This duality is also evident in nitrogen-functionalized activated carbons, where glycine-induced nitrogen groups significantly change surface polarity, adsorption behavior, and long-term material consistency [166].

Recent work from 2020–2025 illustrates both the advantages and potential stability trade-offs in energy storage systems. Nitrogen-doped graphene, CNWs, and other doped carbons often show improved initial electrochemical performance due to enhanced conductivity and redox activity. Examples include N-doped graphene electrodes achieving superior capacity retention in lithium-ion batteries [167], as well as N-doped graphene coatings that stabilize zinc anodes by suppressing dendrite formation and parasitic reactions [168]. These cases demonstrate that heteroatom doping can, under optimal conditions, reinforce long-term cycling stability by improving the solid electrolyte interface and supporting ion transport.

In electrocatalysis, the long-term stability of doped carbon materials is closely linked to the specific configuration of the incorporated heteroatoms. Studies consistently show that edge-type nitrogen species, such as pyridinic and pyrrolic N, provide high initial activity for reactions like the oxygen reduction reaction, but may be more susceptible to structural or chemical changes under extended electrochemical operation [169]. By contrast, graphitic nitrogen, in which N substitutes directly into the carbon lattice, is generally regarded as the more thermodynamically stable configuration, although it typically contributes less to intrinsic catalytic activity. Recent investigations also indicate that introducing heteroatoms inevitably generates lattice strain or defect-rich domains that may accelerate carbon corrosion, microcrack formation, or oxidative degradation during cycling. Similar structure-dependent stability trends have been reported in porous carbon-MoS_2_ composites derived from agricultural biomass, where defect density, graphitization level, and pore architecture critically govern mechanical and electromagnetic robustness [170]. Although substitutional dopants in high-quality CVD-grown graphene or CNTs generally maintain thermal stability, their behavior under repetitive electrochemical cycling is more complex and may involve gradual framework reorganization or localized degradation.

## 15. Conclusions and Outlook

The synthesis science of CVD-derived carbon nanostructures has advanced significantly, yet establishing direct connections between structural features and device performance remains an important challenge. Progress in this field requires not only improved precision in controlling gas phase chemistry, plasma environments, and catalyst or template states, but also a stronger integration between growth conditions and functional outputs. As research moves toward predictive growth control, the ability to tune deposition parameters in real time will allow the deliberate creation of targeted morphologies and properties. Advancing this capability is essential for bridging nanoscale architecture with electrochemical, catalytic, and optoelectronic responses across a broad range of technologies, including sensing, catalysis, energy storage, and photonic applications.

Table 4, provide a clear and systematic overview of the major synthesis determinants, a comparative matrix summarizing the dominant growth regimes and high-priority. CVD control parameters for 0D, 1D, 2D, and 3D carbon nanostructures is presented below. This unified map distills the essential chemical and physical levers that define morphology evolution, ranging from supersaturation and surface diffusion to radical flux and template geometry.

In the case of graphene and CQDs, achieving uniform size distributions, controlled edge chemistry, and stable photoluminescence remains an ongoing challenge. Their optical behavior arises from the combined influence of gas-phase reactions, plasma power, and substrate interactions, which remain insufficiently quantified. Integrating in situ spectroscopy with atomistic modeling can reveal the adatom dynamics that govern emission behavior. Recent plasma-assisted quantum dot studies further show that precursor concentration, quench rate, and hydrogen radical density are critical for attaining narrow size distributions and high crystallinity, findings that align with machine learning-based PECVD models in which gas flow and plasma conditions were identified as the most influential deposition parameters [172].

CNOs offer excellent stability and tunable surface chemistry, yet precise control of shell number, defect content, and dopant distribution remains limited. Temperature-programmed CVD and template-assisted confinement strategies appear particularly promising for refining shell architecture. Insights from atomistic and experimental investigations further show that shell closure and graphitization depend strongly on transformation temperature, dwell time, and precursor curvature, which collectively dictate carbon rearrangement and shell stacking order [173].

CVD-grown NDs provide a robust platform for controlling sp three bonded carbon, although challenges persist in stabilizing small seeds and in suppressing re nucleation under hydrogen-rich environments. Incorporating quantum-relevant color centers such as NV, SiV, or GeV into NDs requires careful control of radical chemistry, oxygen-assisted plasma conditions, and substrate temperature. Machine learning-assisted plasma diagnostics and interpretable deposition rate models have recently demonstrated that methane and hydrogen flows, chamber pressure, and RF power are the key factors that govern diamond film growth, offering a rational foundation for optimizing color-center incorporation [172].

GNRs face dual challenges involving edge chirality and width uniformity. Maintaining well defined armchair or zigzag edges across large areas is hindered by thermal fluctuations and the loss of anisotropic growth conditions. Although vicinal substrates, molecular seeding, and plasma-modulated anisotropic etching strategies offer partial improvement, substantial progress will depend on in situ monitoring of edge reconstruction supported by machine learning models that can predict ribbon width and defect evolution from real-time process data. This strategy aligns with recent machine learning applications in the growth of 2D materials, where temperature, precursor flux, and substrate activation were quantitatively linked to final morphology [174].

For CNWs, the need to balance low-temperature growth with high crystallinity and strong adhesion remains a central challenge. The temperature defect trade-off inherent to plasma enhanced CVD directly impacts conductivity and mechanical robustness. Multi-stage plasma sequencing, real-time plasma diagnostics, and careful control of the CH_4_ to H_2_ ratio, chamber pressure, radical flux, and substrate bias are essential for controlling wall spacing, vertical alignment, and defect density. These controls are strongly supported by detailed mechanistic PECVD analyses that describe nucleation, lifting, and wall thickening as interconnected stages [173].

GFs, produced through template-constrained CVD, demonstrate exceptional electrical and mechanical properties but continue to face challenges related to scalability, template uniformity, and catalyst recycling. Controlling template geometry, precursor flow, and cooling rate is crucial to achieving reproducible porosity and connectivity. These principles are consistent with recent data-driven studies showing that geometric input features significantly affect CVD outcomes in three-dimensional architectures [174].

Across all carbon dimensionalities, meaningful progress will rely on combining precise process control with integrated functional design. Hybrid fabrication strategies that couple CVD with complementary vapor-phase and solution-based processes such as CVT, ALD, and sol–gel synthesis offer pathways to bridge atomic-scale control with scalable engineering. Advances in machine learning-based optimization, including Bayesian process control, interpretable PECVD models, XGBoost process prediction, and data-driven growth mapping, provide powerful tools for navigating complex parameter spaces and enabling reproducible morphology-specific synthesis [172]. Leveraging automation and artificial intelligence, CVD may ultimately transition from an empirical technique to a predictive, high-throughput materials platform capable of delivering carbon nanostructures suitable for industrial and technological deployment.

## Figures and Tables

**Table 1:** Overview of CVD-generated hollow and core–shell carbon nanostructures.

Catalyst or Template Type	Carbon Source (Feedstock)	Temperature (°C)	Morphology/Product	Mechanistic Highlights	Ref.
Ni/Al_2_O_3_ (reduced NiO)	CH_4_ + H_2_	600	Hollow CNOs (~50 nm)	Carbon dissolution–precipitation; Kirkendall voiding	[63]
Co on MgO (1–10 wt%)	C_2_H_4_ + Ar	700	Hollow onions (10–50 nm, 99% purity)	Co decomposes C_2_H_4_; MgO prevents sintering	[62]
MgO, Al_2_O_3_, TiO_2_	C_2_H_5_OH	700–800	Few-layer graphene shells (3–7 layers)	Oxide templating; surface oxygen enables functionalization	[64]
MgO nanospheres	CH_4_ + H_2_	800	Hollow graphitic spheres	MgO-templated graphitization; durable electrodes	[66]
SiO_2_ or Au@SiO_2_	CH_4_	700–900	Hollow or core–shell spheres	Geometric templating via SiO_2_; HF removal	[67]
SiO_2_/AAO templates	Polyphenol vapor	500–950 (ramp)	Hollow carbons (porous)	Non-isothermal CVD; shell thickness control	[68]
SiO_2_ nanoparticles	CH_4_	1000	SiC/graphene core–shell	Partial SiO_2_ → SiC; oxide-carbide transition	[69]
Si nanoparticles + vertical graphene	CVD carbon + H_2_	700–800	Core–shell Si-C	Vertically aligned graphene improves conductivity	[70]
SiO_2_ template + CVD carbon	C_2_H_2_	750	Core–shell Si-C composite	Controlled voids buffer Si expansion	[71]
Metal-free (self-template)	CVD carbon precursor	700	Al-doped hollow cages	Atomic Al doping tunes Li^+^ intercalation	[72]
Dolomite template	CVD + Triphenylphosphine	700	P-doped hollow spheres	P creates electron-rich redox sites	[73]
Dolomite + N-source	CVD + NH_3_	750	N-doped hollow carbons	Multiscale porosity; improved ion transport	[74]

**Table 2:** Overview of key Raman, XPS, and XRD signatures associated with carbon nanomaterials of different dimensionalities.

Carbon Dimension	Representative Materials	Raman Signature	XPS Signature	XRD Signature	Ref.
0D	CQDs, NDs, CNOs, GFs	CQDs: D & G present; broad bands from disorder; weak 2D. NDs: 1332 cm^−1^ diamond peak. CNOs: strong D, broadened G (curved shells).	CQDs: mixed sp^2^/sp^3^ + C–O, C=O, COOH groups. CNOs: mostly sp^2^, graphitization increases with annealing. NDs: dominant sp^3^ core.	CQDs: very broad (002). CNOs: broadened graphitic (002), ~0.34 nm spacing. NDs: sharp diamond reflections (111 at ~44°).	[137,138,141,142,144,150]
1D	Graphene Nanoribbons (GNRs), CNTs	GNRs: strong edge D; RLBM 200–400 cm^−1^; width-dependent features. D-band dispersion is a GNR fingerprint.	Predominantly sp^2^; edges host O/N dopants; chemistry varies with fabrication.	Individual ribbons: no strong (002). Bundled GNRs or CNT-unzipped products show turbostratic (002) features.	[139,145,146]
2D	Graphene Sheets, Carbon Nanowalls (CNWs)	CNWs: strong D (edge density), G prominent. D-band energy dispersion ~46 cm^−1^/eV.	Mostly sp^2^ with small sp^3^; oxygen functional groups present. Valence band resembles graphite.	~0.34 nm layer spacing (graphite-like). Broad (002) due to vertical nanosheet geometry.	[147,148]
3D	Graphene Foams, Aerogels, sp^2^–sp^3^ Graphitic Networks	D and G clear; 2D broad single component; crystallinity improves with annealing.	Predominantly sp^2^; sp^3^ fraction depends on synthesis; annealing reduces oxygen.	Broad (002) that sharpens with wall thickening; sp^2^/sp^3^ hybrids show expanded spacing + diamond + graphitic peaks.	[148,150]

**Table 3:** Summary of major synthesis methods for carbon-based nanomaterials, highlighting differences in process conditions, advantages, limitations and suitability for industrial integration.

Synthesis Method	Typical Products	Key Advantages	Key Limitations	Industrial Scalability Outlook	Ref.
CVD	Graphene, CNTs, CNWs, GNRs, GQDs, NDs, graphitic foams	Precise morphology control; tunable gas chemistry; compatibility with wafer-scale substrates; direct growth on device surfaces; high structural uniformity; plasma-enabled low-T synthesis	High gas consumption; moderate throughput; requires temperature management; sensitive to precursor ratios	High potential: already used in roll-to-roll graphene film production; adaptable to CMOS-compatible low-T PECVD; strong pathway to industrial adoption	[12,101,153,159,160]
Arc Discharge	CNTs, fullerenes, CNOs	High crystallinity; simple apparatus; good for bulk powder production	Poor morphology control; limited tunability; high temperatures; not substrate-compatible	Moderate, but mostly for bulk powders; unsuitable for device integration	[12]
Hydrothermal/Solvothermal	CQDs, CNTs,	Scalable; low temperature; solution processable; easy doping	Poor crystallinity; surface-state-dominated PL; difficult to integrate with solid-state devices	High for inks and coatings; low for device-grade graphitic materials	[161]
Electrochemical Exfoliation	GQDs, CNTs,	Room-temperature; fast; high yield	Produces defected or oxidized structures; broad size distribution	Medium, largely for inks and composites	[162]
Template-Assisted Pyrolysis	GQDs, CNTs,	Good morphological replication; tunable geometry	Requires template removal; limited crystallinity; slower throughput	Medium depending on template cost and manufacturing process	[163]

**Table 4:** Systematic overview of key synthesis determinants for major carbon nanostructures.

Carbon Structure	Dominant Mechanism	Critical Parameters	Typical Ranges	Ref.
0D CQDs, CNOs, and NDs	nucleation-dominated growth driven by high supersaturation	precursor concentration, quench rate, hydrogen radical density	450 to 700 °C, high CH_4_ fraction, strong H radical flux	[63]
1D GNRss	anisotropic surface diffusion and controlled edge reconstruction	temperature uniformity, precursor flux, surface anisotropy, hydrogen concentration	900 to 950 °C, in H_2_/CH_4_ environment	[102,106]
2D CNWss	surface-limited vertical nucleation and radical-assisted graphene sheet lifting	substrate temperature, plasma power, CH_4_ to H_2_ ratio, chamber pressure, radical flux, substrate bias	550 to 750 °C, CH_4_ to H_2_ ratio 1 to 20, 1 to 30 Torr, 200 to 800 watts	[117,118,120,125,171]
GFs	template-constrained conformal deposition and diffusion-driven carbon restructuring	template geometry, pore size, carbon precursor flow, cooling rate	800 to 1000 °C on Ni or Cu foam templates	[126,130]

## Data Availability

No new data were created or analyzed in this study. Data sharing is not applicable to this article.
